# Supplementary material for: SNCA-targeted transcriptional repression therapy alleviates pathological and behavioral perturbations in a Parkinson's disease mouse model
Source: Neurotherapeutics. 2026 Aug 18;23(5):e01049. doi: 10.1016/j.neurot.2026.e01049 (PMC13520098; doi:10.1016/j.neurot.2026.e01049)
Supplement: Supplementary file 1 [file mmc1.pdf]

Table. S1. Complete blood count and serum chemistry data

Parameter ($x10^3$)	Saline (mean±SEM)		LV Repressor (mean±SEM)		LV control (mean±SEM)	
	Male	Female	Male	Female	Male	Female
**Total WBCs**	1.9 ± 0.2	0.85 ± 0.12	0.69 ± 0.15	0.69 ± 0.14	0.86 ± 0.17	0.63 ± 0.12
**Neutrophils**	0.2 ± 0.04	0.1 ± 0.02	0.06 ± 0.02	0.08 ± 0.02	0.12 ± 0.03	0.8 ± 0.01
**Lymphocyte**	1.6 ± 0.19	0.66 ± 0.12	0.59 ± 0.13	0.4 ± 0.12	0.7 ± 0.16	0.52 ± 0.1
**Monocytes**	0.06 ± 0.01	0.4 ± 0.01	0.02 ± 0.01	0.02 ± 0.01	0.03 ± 0.01	0.03 ± 0.01
**Eosinophils**	0.02 ± 0.005	0.01 ± 0.005	0.01 ± 0.005	0.002 ± 0.002	0.007 ± 0.003	0.007 ± 0.005
**Basophils**	0.002 ± 0.001	0.003 ± 0.002	0	0	0	0
**RBCs**	9.7 ± 0.06	8.6 ± 0.36	9.6 ± 0.14	8.7 ± 0.17	9.3 ± 0.24	9.4 ± 0.12
**Platelets**	1279 ± 149	1110 ± 154	1119 ± 241	970 ± 196	1221 ± 155	909 ± 72
**Hematocrit**	42.3 ± 0.35	42.05 ± 0.55	40.8 ± 0.55	40.7 ± 0.52	40.58 ± 0.95	40.58 ± 0.48
**Hemoglobin**	13.79 ± 0.1	13.75 ± 0.17	13.52 ± 0.15	13.57 ± 0.19	13.22 ± 0.24	13.4 ± 0.11
**MCV**	43.6 ± 0.12	43.7 ± 0.25	42.68 ± 1.08	44.8 ± 0.25	43.58 ± 0.38	43.38 ± 0.27
**ALP**	52.77 ± 1.19	71.67 ± 3.58	58.6 ± 8.63	75.83 ± 4.25	51.17 ± 2.74	73.3 ± 4.23
**AST**	65.89 ± 6.79	67.5 ± 10.99	68.6 ± 15.35	61.17 ± 6.54	51.5 ± 7.2	68.67 ± 7.86
**ALT**	7.22 ± 0.64	5.5 ± 0.85	7.8 ± 2.52	5 ± 0.58	4.33 ± 0.42	4.5 ± 0.43
**BUN**	26.78 ± 1.12	29.17 ± 0.19	29.8 ± 2.56	26.17 ± 0.6	25.17 ± 0.95	24.33 ± 0.67
**Total protein**	4.5 ± 0.04	4.33 ± 0.13	4.32 ± 0.89	4.08 ± 0.09	4.35 ± 0.06	4.03 ± 0.07

Supplementary Note: Plasmid Sequences

1. pBK1838 pAAV-CMV-SNCAintron1-AlphaSyn[A53T]-WPRE-SV40pA (PD-INDUCER)

cctgcaggcagctgcgcgctcgctcgctcactgaggccgcccgggcgtcgggcgacctttggtcgcccggcctcagtgagcgagcgag
cgcgcagagagggagtggccaactccatcactaggggttcctgcggccgcacgcgtATcgataagcttgggagttccgcgttacataact
tacggtaaatggcccgcctggctgaccgcccaacgacccccgcccattgacgtcaataatgacgtatgttcccatagtaacgccaataggg
actttccattgacgtcaatgggtggagtatttacggtaaactgcccacttggcagtacatcaagtgtatcatatgccaagtacgccccctattga
cgtcaatgacggtaaatggcccgcctggcattatgcccagtacatgaccttatgggactttcctacttggcagtacatctacgtattagtcatcg
ctattaccatggtgatgcggttttggcagtacatcaatgggcgtggatagcggtttgactcacggggatttccaagtctccaccccattgacgt
caatgggagtttgttttggcaccaaaatcaacgggactttccaaaatgtcgtaacaactccgccccattgacgcaaatgggcggtaggcgtgt
acggtgggaggtctatataagcagagctctctggctaactagagaacccactgcttactggcttatcgaaattaatacgactcactataggga
gacccaagctggCTAGCcgggcgtctttggaaatcctggagaacgccggatgggagacgaatggtcgtgggcaccgggaggggt
ggtgctgccatgaggacccgctgggccaggtctctgggaggtgagtacttgtccctttggggagcctaaggaaagagacttgacctggctt
tcgtcctgcttctgatattcccttctccacaagggctgagagattaggctgcttctccgggatccgcttttccccgggaaacgcgaggatgctc
catggagcgtgagcatccaacttttctctcacataaaatctgtctgcccgctctcttggtttttctctgtaaagtaagcaagctgcgtttggcaaat
aatgaaatggaagtgcaaggaggccaagtcaacaggtggtaacgggttaacaagtgctggcgcggggtccgctagggtggaggctgag
aacgccccctcgggtggctggcgcggggttggagacggcccgcgagtgtgagcggcgcctgctcagggtagatagctgagggcggCt
cgagctcaagcttcgatggatgtattcatgaaaggactttcaaaggccaaggagggagttgtggctgctgctgagaaaaccaaacagggtg
tggcagaagcagcaggaaagacaaaagagggtgttctctatgtaggctccaaaaccaaggagggagtggtgcatggtgtgacaacagtg
gctgagaagaccaaagagcaagtgacaaatgttggaggagcagtggtgacgggtgtgacagcagtagcccagaagacagtggaggga
gcagggagcattgcagcagccactggctttgtcaaaaaggaccagttgggcaagaatgaagaaggagccccacaggaaggaattctgga
agatatgcctgtggatcctgacaatgaggcttatgaaatgccttctgaggaagggtatcaagactacgaacctgaagcctaagaattctgcag
tcgagcgggatcaattccgccccccccctaacgttactggccgaagccgcttggaataaggccggtgtgcgtttgtctatatgttattttccac
catattgccgtcttttggcaatgtgagggcccggaaacctggccctgtcttcttgacgagcattcctaggggtctttcccctctcgccaaagga
atgcaaggtctgttgaatgtcgtgaaggaagcagttcctctggaagcttcttgaagacaaacaacgtctgtagcgaccctttgcaggcagcg
gaaccccccacctggcgacaggtgcctctgcggccaaaagccacgtgtataagatacacctgcaaaggcggcacaaccccagtgccac
gttgtgagttggatagttgtggaaagagtcaaatggctctcctcaagcgtattcaacaaggggctgaaggatgcccagaaggtacaagtaag
cGGCCGCTccggaatcaacctctggattacaaaatttgtgaaagattgactggtattcttaactatgttgctccttttacgctatgtggatac
gctgctttaatgcctttgtatcatgctattgcttcccgtatggctttcattttctcctccttgtataaatcctggttgctgtctctttatgaggagttgtgg
cccgttgtcaggcaacgtggcgtggtgtgcactgtgtttgctgacgcaacccccactggttggggcattgccaccacctgtcagctcctttcc
gggactttcgctttccccctccctattgccacggcggaactcatcgccgcctgccttgcccgctgctggacaggggctcggctgttgggcac
tgacaattccgtggtgttgtcggggaagctgacgtcctttccatggctgctcgcctgtgttgccacctggattctgcgcgggacgtccttctgct
acgtcccttcggccctcaatccagcggaccttccttcccgcggcctgctgccggctctgcggcctcttccgcgtcttcgccttcgccctcaga
cgagtcggatctccctttgggccgcctccccgcctgGATCCgtcgacccgggcggccgcttcgagcagacatgataagatacattgat
gagtttggacaaaccacaactagaatgcagtgaaaaaaatgctttatttgtgaaatttgtgatgctattgctttatttgtaaccattataagctgca
ataaacaagttaacaacaacaattgcattcattttatgtttcaggttcagggggagatgtgggaggttttttaaaGgtaaccacgtgcggaccg
agcggccgcaggaacccctagtgatggagttggccactccctctctgcgcgctcgctcgctcactgaggccgggcgaccaaaggtcgcc
cgacgcccgggctttgcccgggcggcctcagtgagcgagcgagcgcgcagctgcctgcaggggcgcctgatgcggtattttctccttacg
catctgtgcggtatttcacaccgcatacgtcaaagcaaccatagtacgcgccctgtagcggcgcattaagcgcggcgggtgtggtggttac
gcgcagcgtgaccgctacacttgccagcgccttagcgcccgctcctttcgctttcttcccttcctttctcgccacgttcgccggctttccccgtc

aagctctaaatcgggggctccctttagggttccgatttagtgctttacggcacctcgaccccaaaaaacttgatttgggtgatggttcacgtagt
gggccatcgccctgatagacggtttttcgccctttgacgttggagtccacgttctttaatagtggactcttgttccaaactggaacaacactcaa
ctctatctcgggctattcttttgatttataagggattttgccgatttcggtctattggttaaaaaatgagctgatttaacaaaaatttaacgcgaatttt
aacaaaatattaacgtttacaattttatggtgcactctcagtacaatctgctctgatgccgcatagttaagccagccccgacacccgccaacac
ccgctgacgcgccctgacgggcttgtctgctcccggcatccgcttacagacaagctgtgaccgtctccgggagctgcatgtgtcagaggttt
tcaccgtcatcaccgaaacgcgcgagacgaaagggcctcgtgatacgcctatttttataggttaatgtcatgataataatggtttcttagacgtc
aggtggcacttttcggggaaatgtgcgcggaacccctatttgtttatttttctaaatacattcaaatatgtatccgctcatgagacaataaccctga
taaatgcttcaataatattgaaaaaggaagagtatgagtattcaacatttccgtgtcgcccttattcccttttttgcggcattttgccttcctgtttttg
ctcacccagaaacgctggtgaaagtaaaagatgctgaagatcagttgggtgcacgagtgggttacatcgaactggatctcaacagcggtaa
gatccttgagagttttcgccccgaagaacgttttccaatgatgagcacttttaaagttctgctatgtggcgcggtattatcccgtattgacgccgg
gcaagagcaactcggtcgccgcatacactattctcagaatgacttggttgagtactcaccagtcacagaaaagcatcttacggatggcatga
cagtaagagaattatgcagtgctgccataaccatgagtgataacactgcggccaacttacttctgacaacgatcggaggaccgaaggagct
aaccgcttttttgcacaacatgggggatcatgtaactcgccttgatcgttgggaaccggagctgaatgaagccataccaaacgacgagcgtg
acaccacgatgcctgtagcaatggcaacaacgttgcgcaaactattaactggcgaactacttactctagcttcccggcaacaattaatagact
ggatggaggcggataaagttgcaggaccacttctgcgctcggcccttccggctggctggtttattgctgataaatctggagccggtgagcgt
gggtctcgcggtatcattgcagcactggggccagatggtaagccctcccgtatcgtagttatctacacgacggggagtcaggcaactatgg
atgaacgaaatagacagatcgctgagataggtgcctcactgattaagcattggtaactgtcagaccaagtttactcatatatactttagattgatt
taaaacttcatttttaatttaaaaggatctaggtgaagatcctttttgataatctcatgaccaaaatcccttaacgtgagttttcgttccactgagcgt
cagaccccgtagaaaagatcaaaggatcttcttgagatcctttttttctgcgcgtaatctgctgcttgcaaacaaaaaaaccaccgctaccagc
ggtggtttgtttgccggatcaagagctaccaactctttttccgaaggtaactggcttcagcagagcgcagataccaaatactgttcttctagtgt
agccgtagttaggccaccacttcaagaactctgtagcaccgcctacatacctcgctctgctaatcctgttaccagtggctgctgccagtggcg
ataagtcgtgtcttaccgggttggactcaagacgatagttaccggataaggcgcagcggtcgggctgaacggggggttcgtgcacacagc
ccagcttggagcgaacgacctacaccgaactgagatacctacagcgtgagctatgagaaagcgccacgcttcccgaagggagaaaggc
ggacaggtatccggtaagcggcagggtcggaacaggagagcgcacgagggagcttccagggggaaacgcctggtatctttatagtcctg
tcgggtttcgccacctctgacttgagcgtcgatttttgtgatgctcgtcaggggggcggagcctatggaaaaacgccagcaacgcggcctttt
tacggttcctggccttttgctggccttttgctcacatgt

2. **pBK1132- Lenti-EFSNC-dSaCas9-U6-SAgRNA scaffold (INACTIVE VECTOR, no gRNA, no repressor)**

gcacttttcggggaaatgtgcgcggaacccctatttgtttatttttctaaatacattcaaatatgtatccgctcatgagacaataaccctgataaat
gcttcaataatattgaaaaaggaagagtatgagtattcaacatttccgtgtcgcccttattcccttttttgcggcattttgccttcctgtttttgctcac
ccagaaacgctggtgaaagtaaaagatgctgaagatcagttgggtgcacgagtgggttacatcgaactggatctcaacagcggtaagatcc
ttgagagttttcgccccgaagaacgttttccaatgatgagcacttttaaagttctgctatgtggcgcggtattatcccgtattgacgccgggcaa
gagcaactcggtcgccgcatacactattctcagaatgacttggttgagtactcaccagtcacagaaaagcatcttacggatggcatgacagta
agagaattatgcagtgctgccataaccatgagtgataacactgcggccaacttacttctgacaacgatcggaggaccgaaggagctaaccg
cttttttgcacaacatgggggatcatgtaactcgccttgatcgttgggaaccggagctgaatgaagccataccaaacgacgagcgtgacacc
acgatgcctgtagcaatggcaacaacgttgcgcaaactattaactggcgaactacttactctagcttcccggcaacaattaatagactggatg
gaggcggataaagttgcaggaccacttctgcgctcggcccttccggctggctggtttattgctgataaatctggagccggtgagcgtgggtc
tcgcggtatcattgcagcactggggccagatggtaagccctcccgtatcgtagttatctacacgacggggagtcaggcaactatggatgaa

cgaaatagacagatcgctgagataggtgcctcactgattaagcattggtaactgtcagaccaagtttactcatatatactttagattgatttaaaa
cttcatttttaatttaaaaggatctaggtgaagatcctttttgataatctcatgaccaaaatcccttaacgtgagttttcgttccactgagcgtcaga
ccccgtagaaaagatcaaaggatcttcttgagatcctttttttctgcgcgtaatctgctgcttgcaaacaaaaaaaccaccgctaccagcggtg
gtttgtttgccggatcaagagctaccaactctttttccgaaggtaactggcttcagcagagcgcagataccaaatactgttcttctagtgtagcc
gtagttaggccaccacttcaagaactctgtagcaccgcctacatacctcgctctgctaatcctgttaccagtggctgctgccagtggcgataa
gtcgtgtcttaccgggttggactcaagacgatagttaccggataaggcgcagcggtcgggctgaacggggggttcgtgcacacagcccag
cttggagcgaacgacctacaccgaactgagatacctacagcgtgagctatgagaaagcgccacgcttcccgaagggagaaaggcggac
aggtatccggtaagcggcagggtcggaacaggagagcgcacgagggagcttccagggggaaacgcctggtatctttatagtcctgtcgg
gtttcgccacctctgacttgagcgtcgatttttgtgatgctcgtcaggggggcggagcctatggaaaaacgccagcaacgcggcctttttacg
gttcctggccttttgctggccttttgctcacatgttctttcctgcgttatcccctgattctgtggataaccgtattaccgcctttgagtgagctgatac
cgctcgccgcagccgaacgaccgagcgcagcgagtcagtgagcgaggaagcggaagagcgcccaatacgcaaaccgcctctccccg
cgcgttggccgattcattaatgcagctggcacgacaggtttcccgactggaaagcgggcagtgagcgcaacgcaattaatgtgagttagct
cactcattaggcaccccaggctttacactttatgcttccggctcgtatgttgtgtggaattgtgagcggataacaatttcacacaggaaacagct
atgaccatgattacgccaagcgcgcaattaaccctcactaaagggaacaaaagctggagctgcaagcttaatgtagtcttatgcaatactctt
gtagtcttgcaacatggtaacgatgagttagcaacatgccttacaaggagagaaaaagcaccgtgcatgccgattggtggaagtaaggtgg
tacgatcgtgccttattaggaaggcaacagacgggtctgacatggattggacgaaccactgaattgccgcattgcagagatattgtatttaagt
gcctagctcgatacataaacgggtctctctggttagaccagatctgagcctgggagctctctggctaactagggaacccactgcttaagcctc
aataaagcttgccttgagtgcttcaagtagtgtgtgcccgtctgttgtgtgactctggtaactagagatccctcagacccttttagtcagtgtgga
aaatctctagcagtggcgcccgaacagggacttgaaagcgaaagggaaaccagaggagctctctcgacgcaggactcggcttgctgaag
cgcgcacggcaagaggcgaggggcggcgactggtgagtacgccaaaaattttgactagcggaggctagaaggagagagatgggtgcg
agagcgtcagtattaagcgggggagaattagatcgcgatgggaaaaaattcggttaaggccagggggaaagaaaaaatataaattaaaac
atatagtatgggcaagcagggagctagaacgattcgcagttaatcctggcctgttagaaacatcagaaggctgtagacaaatactgggaca
gctacaaccatcccttcagacaggatcagaagaacttagatcattatataatacagtagcaaccctctattgtgtgcatcaaaggatagagata
aaagacaccaaggaagctttagacaagatagaggaagagcaaaacaaaagtaagaccaccgcacagcaagcggccgctgatcttcaga
cctggaggaggagatatgagggacaattggagaagtgaattatataaatataaagtagtaaaaattgaaccattaggagtagcacccaccaa
ggcaaagagaagagtggtgcagagagaaaaaagagcagtgggaataggagctttgttccttgggttcttgggagcagcaggaagcactat
gggcgcagcgtcaatgacgctgacggtacaggccagacaattattgtctggtatagtgcagcagcagaacaatttgctgagggctattgag
gcgcaacagcatctgttgcaactcacagtctggggcatcaagcagctccaggcaagaatcctggctgtggaaagatacctaaaggatcaa
cagctcctggggatttggggttgctctggaaaactcatttgcaccactgctgtgccttggaatgctagttggagtaataaatctctggaacagat
ttggaatcacacgacctggatggagtgggacagagaaattaacaattacacaagcttaatacactccttaattgaagaatcgcaaaaccagc
aagaaaagaatgaacaagaattattggaattagataaatgggcaagtttgtggaattggtttaacataacaaattggctgtggtatataaaattat
tcataatgatagtaggaggcttggtaggtttaagaatagtttttgctgtactttctatagtgaatagagttaggcagggatattcaccattatcgttt
cagacccacctcccaaccccgaggggacccgacaggcccgaaggaatagaagaagaaggtggagagagagacagagacagatccat
tcgattagtgaacggatctcgacggtatcggttaacttttaaaagaaaaggggggattggggggtacagtgcaggggaaagaatagtagac
ataatagcaacagacatacaaactaaagaattacaaaaacaaattacaaaattcaaaattttatcgGcttgaaaggagtgggaattggctccg
gtgcccgtcagtgggcagagcgcacatcgcccacagtccccgagaagttggggggaggggtcggcaattgatccggtgcctagagaag
gtggcgcggggtaaactgggaaagtgatgtcgtgtactggctccgcctttttcccgagggtgggggagaaccgtatataagtgcagtagtc
gccgtgaacgttctttttcgcaacgggtttgccgccagaacacaggaccggtgccaccatgGACTACAAAGACCATGACG
GTGATTATAAAGATCATGACATaGATTACAAGGATGACGATGACAAGggtggatcgccaaagaaa
aagaggaaagtcgccccaaagaagaagcggaaggtcggtatccacggagtcccagcagccaagcggaactacatcctgggcctggcc

atcggcatcaccagcgtgggctacggcatcatcgactacgagacacgggacgtgatcgatgccggcgtgcggctgttcaaagaggccaa
cgtggaaaacaacgagggcaggcggagcaagagaggcgccagaaggctgaagcggcggaggcggcatagaatccagagagtgaag
aagctgctgttcgactacaacctgctgaccgaccacagcgagctgagcggcatcaacccctacgaggccagagtgaagggcctgagcca
gaagctgagcgaggaagagttctctgccgccctgctgcacctggccaagagaagaggcgtgcacaacgtgaacgaggtggaagagga
caccggcaacgagctgtccaccaaagagcagatcagccggaacagcaaggccctggaagagaaatacgtggccgaactgcagctgga
acggctgaagaaagacggcgaagtgcggggcagcatcaacagattcaagaccagcgactacgtgaaagaagccaaacagctgctgaa
ggtgcagaaggcctaccaccagctggaccagagcttcatcgacacctacatcgacctgctggaaacccggcggacctactatgagggac
ctggcgagggcagccccttcggctggaaggacatcaaagaatggtacgagatgctgatgggccactgcacctacttccccgaggaactg
cggagcgtgaagtacgcctacaacgccgacctgtacaacgccctgaacgacctgaacaatctcgtgatcaccagggacgagaacgagaa
gctggaatattacgagaagttccagatcatcgagaacgtgttcaagcagaagaagaagcccaccctgaagcagatcgccaaagaaatcct
cgtgaacgaagaggatattaagggctacagagtgaccagcaccggcaagcccgagttcaccaacctgaaggtgtaccacgacatcaagg
acattaccgcccggaaagagattattgagaacgccgagctgctggatcagattgccaagatcctgaccatctaccagagcagcgaggaca
tccaggaagaactgaccaatctgaactccgagctgacccaggaagagatcgagcagatctctaatctgaagggctataccggcacccaca
acctgagcctgaaggccatcaacctgatcctggacgagctgtggcacaccaacgacaaccagatcgctatcttcaaccggctgaagctggt
gcccaagaaggtggacctgtcccagcagaaagagatccccaccaccctggtggacgacttcatcctgagccccgtcgtgaagagaagctt
catccagagcatcaaagtgatcaacgccatcatcaagaagtacggcctgcccaacgacatcattatcgagctggcccgcgagaagaactc
caaggacgcccagaaaatgatcaacgagatgcagaagcggaaccggcagaccaacgagcggatcgaggaaatcatccggaccaccg
gcaaagagaacgccaagtacctgatcgagaagatcaagctgcacgacatgcaggaaggcaagtgcctgtacagcctggaagccatccct
ctggaagatctgctgaacaaccccttcaactatgaggtggaccacatcatccccagaagcgtgtccttcgacaacagcttcaacaacaaggt
gctcgtgaagcaggaagaagccagcaagaagggcaaccggaccccattccagtacctgagcagcagcgacagcaagatcagctacga
aaccttcaagaagcacatcctgaatctggccaagggcaagggcagaatcagcaagaccaagaaagagtatctgctggaagaacgggac
atcaacaggttctccgtgcagaaagacttcatcaaccggaacctggtggataccagatacgccaccagaggcctgatgaacctgctgcgg
agctacttcagagtgaacaacctggacgtgaaagtgaagtccatcaatggcggcttcaccagctttctgcggcggaagtggaagtttaagaa
agagcggaacaaggggtacaagcaccacgccgaggacgccctgatcattgccaacgccgatttcatcttcaaagagtggaagaaactgg
acaaggccaaaaaagtgatggaaaaccagatgttcgaggaaaagcaggccgagagcatgcccgagatcgaaaccgagcaggagtaca
aagagatcttcatcacccccccaccagatcaagcacattaaggacttcaaggactacaagtacagccaccgggtggacaagaagcctaata
gagagctgattaacgacaccctgtactccacccggaaggacgacaagggcaacaccctgatcgtgaacaatctgaacggcctgtacgac
aaggacaatgacaagctgaaaaagctgatcaacaagagccccgaaaagctgctgatgtaccaccacgacccccagacctaccagaaact
gaagctgattatggaacagtacggcgacgagaagaatcccctgtacaagtactacgaggaaaccgggaactacctgaccaagtactccaa
aaaggacaacggccccgtgatcaagaagattaagtattacggcaacaaactgaacgcccatctggacatcaccgacgactaccccaacag
cagaaacaaggtcgtgaagctgtccctgaagccctacagattcgacgtgtacctggacaatggcgtgtacaagttcgtgaccgtgaagaat
ctggatgtgatcaaaaaagaaaactactacgaagtgaatagcaagtgctatgaggaagctaagaagctgaagaagatcagcaaccaggcc
gagtttatcgcctccttctacaacaacgatctgatcaagatcaacggcgagctgtatagagtgatcggcgtgaacaacgacctgctgaaccg
gatcgaagtgaacatgatcgacatcacctaccgcgagtacctggaaaacatgaacgacaagaggccccccaggatcattaagacaatcgc
ctccaagacccagagcattaagaagtacagcacagacattctgggcaacctgtatgaagtgaaatctaagaagcaccctcagatcatcaaa
aagggcaaaaggccggcggccacgaaaaaggccggccaggcaaaaaagaaaaaggGATCCTGAAATAAAAGATCT
TTGTTTTCATTAGATCTGTGTGTTGGTTTTTTGTGTGCggCCggtaccgagggcctatttcccatgattcc
ttcatatttgcatatacgatacaaggctgttagagagataattggaattaatttgactgtaaacacaaagatattagtacaaaatacgtgacgtag
aaagtaataatttcttgggtagtttgcagttttaaaattatgttttaaaatggactatcatatgcttaccgtaacttgaaagtatttcgatttcttggcttt
atatatcttgtggaaaggacgaaacaccggagaccacggcaggtctcagttttagtactctggaaacagaatctactaaaacaaggcaaaat

gccgtgtttatctcgtcaacttgttggcgagattAGCaattcgagctcggtacctttaagaccaatgacttacaaggcagctgtagatcttag
ccactttttaaaagaaaaggggggactggaagggctaattcactcccaacgaagacaagatctgctttttgcttgtactgggtctctctggtta
gaccagatctgagcctgggagctctctggctaactagggaacccactgcttaagcctcaataaagcttgccttgagtgcttcaagtagtgtgt
gcccgtctgttgtgtgactctggtaactagagatccctcagacccttttagtcagtgtggaaaatctctagcagtagtagttcatgtcatcttatta
ttcagtatttataacttgcaaagaaatgaatatcagagagtgagaggaacttgtttattgcagcttataatggttacaaataaagcaatagcatca
caaatttcacaaataaagcatttttttcactgcattctagttgtggtttgtccaaactcatcaatgtatcttatcatgtctggctctagctatcccgccc
ctaactccgcccatcccgcccctaactccgcccagttccgcccattctccgccccatggctgactaattttttttatttatgcagaggccgaggc
cgcctcggcctctgagctattccagaagtagtgaggaggcttttttggaggcctagggacgtacccaattcgccctatagtgagtcgtattac
gcgcgctcactggccgtcgttttacaacgtcgtgactgggaaaaccctggcgttacccaacttaatcgccttgcagcacatccccctttcgcc
agctggcgtaatagcgaagaggcccgcaccgatcgcccttcccaacagttgcgcagcctgaatggcgaatgggacgcgccctgtagcgg
cgcattaagcgcggcgggtgtggtggttacgcgcagcgtgaccgctacacttgccagcgccctagcgcccgctcctttcgctttcttcccttc
ctttctcgccacgttcgccggctttccccgtcaagctctaaatcgggggctccctttagggttccgatttagtgctttacggcacctcgacccca
aaaaacttgattagggtgatggttcacgtagtgggccatcgccctgatagacggtttttcgccctttgacgttggagtccacgttctttaatagtg
gactcttgttccaaactggaacaacactcaaccctatctcggtctattcttttgatttataagggattttgccgatttcggcctattggttaaaaaat
gagctgatttaacaaaaatttaacgcgaattttaacaaaatattaacgcttacaatttaggtg

3. pBK1579- pLV-U6-[empty]-EFSNC-dSaCas9-DNMT3a-P2A-Puro-WPRE (pCL17) (no gRNA vector)

ctagttattaatagtaatcaattacggggtcattagttcatagcccatatatggagttccgcgttacataacttacggtaaatggcccgcctggct
gaccgcccaacgacccccgcccattgacgtcaataatgacgtatgttcccatagtaacgccaatagggactttccattgacgtcaatgggtg
gagtatttacggtaaactgcccacttggcagtacatcaagtgtatcatatgccaagtacgccccctattgacgtcaatgacggtaaatggccc
gcctggcattatgcccagtacatgaccttatgggactttcctacttggcagtacatctacgtattagtcatcgctattaccatggtgatgcggtttt
ggcagtacatcaatgggcgtggatagcggtttgactcacggggatttccaagtctccaccccattgacgtcaatgggagtttgttttggcacc
aaaatcaacgggactttccaaaatgtcgtaacaactccgccccattgacgcaaatgggcggtaggcgtgtacggtgggaggtctatataag
cagcgcgttttgcctgtactgggtctctctggttagaccagatctgagcctgggagctctctggctaactagggaacccactgcttaagcctca
ataaagcttgccttgagtgcttcaagtagtgtgtgcccgtctgttgtgtgactctggtaactagagatccctcagacccttttagtcagtgtggaa
aatctctagcagtggcgcccgaacagggacttgaaagcgaaagggaaaccagaggagctctctcgacgcaggactcggcttgctgaagc
gcgcacggcaagaggcgaggggcggcgactggtgagtacgccaaaaattttgactagcggaggctagaaggagagagatgggtgcga
gagcgtcagtattaagcgggggagaattagatcgcgatgggaaaaaattcggttaaggccagggggaaagaaaaaatataaattaaaaca
tatagtatgggcaagcagggagctagaacgattcgcagttaatcctggcctgttagaaacatcagaaggctgtagacaaatactgggacag
ctacaaccatcccttcagacaggatcagaagaacttagatcattatataatacagtagcaaccctctattgtgtgcatcaaaggatagagataa
aagacaccaaggaagctttagacaagatagaggaagagcaaaacaaaagtaagaccaccgcacagcaagcggccgctgatcttcagac
ctggaggaggagatatgagggacaattggagaagtgaattatataaatataaagtagtaaaaattgaaccattaggagtagcacccaccaa
ggcaaagagaagagtggtgcagagagaaaaaagagcagtgggaataggagctttgttccttgggttcttgggagcagcaggaagcactat
gggcgcagcgtcaatgacgctgacggtacaggccagacaattattgtctggtatagtgcagcagcagaacaatttgctgagggctattgag
gcgcaacagcatctgttgcaactcacagtctggggcatcaagcagctccaggcaagaatcctggctgtggaaagatacctaaaggatcaa
cagctcctggggatttggggttgctctggaaaactcatttgcaccactgctgtgccttggaatgctagttggagtaataaatctctggaacagat
ttggaatcacacgacctggatggagtgggacagagaaattaacaattacacaagcttaatacactccttaattgaagaatcgcaaaaccagc
aagaaaagaatgaacaagaattattggaattagataaatgggcaagtttgtggaattggtttaacataacaaattggctgtggtatataaaattat

tcataatgatagtaggaggcttggtaggtttaagaatagtttttgctgtactttctatagtgaatagagttaggcagggatattcaccattatcgttt
cagacccacctcccaaccccgaggggacccgacaggcccgaaggaatagaagaagaaggtggagagagagacagagacagatccat
tcgattagtgaacggatcggcactgcgtgcgccaattctgcagacaaatggcagtattcatccacaattttaaaagaaaaggggggattggg
gggtacagtgcaggggaaagaatagtagacataatagcaacagacatacaaactaaagaattacaaaaacaaattacaaaaattcaaaattt
tcgggtttattacagggacagcagagatccagtttggTTAATTAATGGCGGGACGTTAACGGGGCGGAAC
GGTACCgagggcctatttcccatgattccttcatatttgcatatacgatacaaggctgttagagagataattagaattaatttgactgtaaac
acaaagatattagtacaaaatacgtgacgtagaaagtaataatttcttgggtagtttgcagttttaaaattatgttttaaaatggactatcatatgctt
accgtaacttgaaagtatttcgatttcttggctttatatatcttGTGGAAAGGACGAAAcaccggagacgtgtacacgtctctgT
TTtagtactctggaaacagaatctactaaaacaaggcaaaatgccgtgtttatctcgtcaacttgttggcgagaTTTTTTgaattcgcta
gctaggtcttgaaaggagtgggaattggctccggtgcccgtcagtgggcagagcgcacatcgcccacagtccccgagaagttggggga
ggggtcggcaattgatccggtgcctagagaaggtggcgcggggtaaactgggaaagtgatgtcgtgtactggctccgcctttttcccgagg
gtgggggagaaccgtatataagtgcagtagtcgccgtgaacgttctttttcgcaacgggtttgccgccagaacacaggaccggtgccacca
tgGACTACAAAGACCATGACGGTGATTATAAAGATCATGACATaGATTACAAGGATGAC
GATGACAAGggtggatcgccaaagaaaaagaggaaagtcgccccaaagaagaagcggaaggtcggtatccacggagtcccag
cagccaagcggaactacatcctgggcctggccatcggcatcaccagcgtgggctacggcatcatcgactacgagacacgggacgtgatc
gatgccggcgtgcggctgttcaaagaggccaacgtggaaaacaacgagggcaggcggagcaagagaggcgccagaaggctgaagcg
gcggaggcggcatagaatccagagagtgaagaagctgctgttcgactacaacctgctgaccgaccacagcgagctgagcggcatcaac
ccctacgaggccagagtgaagggcctgagccagaagctgagcgaggaagagttctctgccgccctgctgcacctggccaagagaagag
gcgtgcacaacgtgaacgaggtggaagaggacaccggcaacgagctgtccaccaaagagcagatcagccggaacagcaaggccctg
gaagagaaatacgtggccgaactgcagctggaacggctgaagaaagacggcgaagtgcggggcagcatcaacagattcaagaccagc
gactacgtgaaagaagccaaacagctgctgaaggtgcagaaggcctaccaccagctggaccagagcttcatcgacacctacatcgacct
gctggaaacccggcggacctactatgagggacctggcgagggcagccccttcggctggaaggacatcaaagaatggtacgagatgctg
atgggccactgcacctacttccccgaggaactgcggagcgtgaagtacgcctacaacgccgacctgtacaacgccctgaacgacctgaa
caatctcgtgatcaccagggacgagaacgagaagctggaatattacgagaagttccagatcatcgagaacgtgttcaagcagaagaagaa
gcccaccctgaagcagatcgccaaagaaatcctcgtgaacgaagaggatattaagggctacagagtgaccagcaccggcaagcccgag
ttcaccaacctgaaggtgtaccacgacatcaaggacattaccgcccggaaagagattattgagaacgccgagctgctggatcagattgcca
agatcctgaccatctaccagagcagcgaggacatccaggaagaactgaccaatctgaactccgagctgacccaggaagagatcgagcag
atctctaatctgaagggctataccggcacccacaacctgagcctgaaggccatcaacctgatcctggacgagctgtggcacaccaacgac
aaccagatcgctatcttcaaccggctgaagctggtgcccaagaaggtggacctgtcccagcagaaagagatccccaccaccctggtggac
gacttcatcctgagccccgtcgtgaagagaagcttcatccagagcatcaaagtgatcaacgccatcatcaagaagtacggcctgcccaacg
acatcattatcgagctggcccgcgagaagaactccaaggacgcccagaaaatgatcaacgagatgcagaagcggaaccggcagaccaa
cgagcggatcgaggaaatcatccggaccaccggcaaagagaacgccaagtacctgatcgagaagatcaagctgcacgacatgcaggaa
ggcaagtgcctgtacagcctggaagccatccctctggaagatctgctgaacaaccccttcaactatgaggtggaccacatcatccccagaa
gcgtgtccttcgacaacagcttcaacaacaaggtgctcgtgaagcaggaagaagccagcaagaagggcaaccggaccccattccagtac
ctgagcagcagcgacagcaagatcagctacgaaaccttcaagaagcacatcctgaatctggccaagggcaagggcagaatcagcaaga
ccaagaaagagtatctgctggaagaacgggacatcaacaggttctccgtgcagaaagacttcatcaaccggaacctggtggataccagat
acgccaccagaggcctgatgaacctgctgcggagctacttcagagtgaacaacctggacgtgaaagtgaagtccatcaatggcggcttca
ccagctttctgcggcggaagtggaagtttaagaaagagcggaacaaggggtacaagcaccacgccgaggacgccctgatcattgccaac
gccgatttcatcttcaaagagtggaagaaactggacaaggccaaaaaagtgatggaaaaccagatgttcgaggaaaagcaggccgagag
catgcccgagatcgaaaccgagcaggagtacaaagagatcttcatcacccccccaccagatcaagcacattaaggacttcaaggactacaa

gtacagccaccgggtggacaagaagcctaatagagagctgattaacgacaccctgtactccacccggaaggacgacaagggcaacacc
ctgatcgtgaacaatctgaacggcctgtacgacaaggacaatgacaagctgaaaaagctgatcaacaagagccccgaaaagctgctgatg
taccaccacgacccccagacctaccagaaactgaagctgattatggaacagtacggcgacgagaagaatcccctgtacaagtactacgag
gaaaccgggaactacctgaccaagtactccaaaaaggacaacggccccgtgatcaagaagattaagtattacggcaacaaactgaacgc
ccatctggacatcaccgacgactaccccaacagcagaaacaaggtcgtgaagctgtccctgaagccctacagattcgacgtgtacctgga
caatggcgtgtacaagttcgtgaccgtgaagaatctggatgtgatcaaaaaagaaaactactacgaagtgaatagcaagtgctatgaggaa
gctaagaagctgaagaagatcagcaaccaggccgagtttatcgcctccttctacaacaacgatctgatcaagatcaacggcgagctgtata
gagtgatcggcgtgaacaacgacctgctgaaccggatcgaagtgaacatgatcgacatcacctaccgcgagtacctggaaaacatgaacg
acaagaggcccccccaggatcattaagacaatcgcctccaagacccagagcattaagaagtacagcacagacattctgggcaacctgtatg
aagtgaaatctaagaagcaccctcagatcatcaaaaagggcaaaaggccggcggccacgaaaaaggcGcTCGAGGGCGGA
GGCGGGAGCGGATCCCCCTCCCGGCTCCAGATGttcttcgctaataaccacgaccaggaatttgaccctccaa
aggtttacccacctgtcccagctgagaagaggaagcccatccgggtgctgtctctctttgatggaatcgctacagggctcctggtgctgaag
gacttgggcattcaggtggaccgctacattgcctcggaggtgtgtgaggactccatcacggtgggcatggtgcggcaccaggggaagatc
atgtacgtcggggacgtccgcagcgtcacacagaagcatatccaggagtggggcccattcgatctggtgattgggggcagtccctgcaat
gacctctccatcgtcaaccctgctcgcaagggcctctacgagggcactggccggctcttctttgagttctaccgcctcctgcatgatgcgcgg
cccaaggagggagatgatcgccccttcttctggctctttgagaatgtggtggccatgggcgttagtgacaagagggacatctcgcgatttctc
gagtccaaccctgtgatgattgatgccaaagaagtgtcagctgcacacagggcccgctacttctggggtaaccttcccggtatgaacaggc
cgttggcatccactgtgaatgataagctggagctgcaggagtgtctggagcatggcaggatagccaagttcagcaaagtgaggaccattac
tacgaggtcaaactccataaagcagggcaaaGACCAGCATTTTCCTGTGTTCATGAATGAGAAAGAGga
catcttatggtgcactgaaatggaaagggtatttggtttcccagtccactatactgacgtgtccaacatgagccgcttggcgaggcagagact
gctgggccggtcatggagcgtgccagtcatccgccacctcttcgctcCGCTGAAGGAGTATTTTGCGTGTGTGtcc
ggccggcccggatccGGCGCAACAAACTTCTCTCTGCTGAAACAAGCCGGAGATGTCGAAG
AGAATCCTGGACCGACCGAGTACAAGCCCACGGTGCGCCTCGCCACCCGCGACGAC
GTCCCCAGGGCCGTACGCACCCTCGCCGCCGCGTTCGCCGACTACCCCGCCACGCGC
CACACCGTCGATCCGGACCGCCACATCGAGCGGGTCACCGAGCTGCAAGAACTCTTC
CTCACGCGCGTCGGGCTCGACATCGGCAAGGTGTGGGTCGCGGACGACGGCGCCGC
GGTGGCGGTCTGGACCACGCCGGAGAGCGTCGAAGCGGGGGCGGTGTTCGCCGAGA
TCGGCCCGCGCATGGCCGAGTTGAGCGGTTCCCGGCTGGCCGCGCAGCAACAGATG
GAAGGCCTCCTGGCGCCGCACCGGCCCAAGGAGCCCGCGTGGTTCCTGGCCACCGT
CGGAGTCTCGCCCGACCACCAGGGCAAGGGTCTGGGCAGCGCCGTCGTGCTCCCCG
GAGTGGAGGCGGCCGAGCGCGCCGGGGTGCCCGCCTTCCTGGAGACCTCCGCGCCC
CGCAACCTCCCCTTCTACGAGCGGCTCGGCTTCACCGTCACCGCCGACGTCGAGGTG
CCCGAAGGACCGCGCACCTGGTGCATGACCCGCAAGCCCGGTGCCTGAACGCGTTA
AGTCGACAATCAACCTCTGGATTACAAAATTTGTGAAAGATTGACTGGTATTCTTAAC
TATGTTGCTCCTTTTACGCTATGTGGATACGCTGCTTTAATGCCTTTGTATCATGCTATT
GCTTCCCGTATGGCTTTCATTTTCTCCTCCTTGTATAAATCCTGGTTGCTGTCTCTTTAT
GAGGAGTTGTGGCCCGTTGTCAGGCAACGTGGCGTGGTGTGCACTGTGTTTGCTGAC
GCAACCCCCACTGGTTGGGGCATTGCCACCACCTGTCAGCTCCTTTCCGGGACTTTC
GCTTTCCCCCTCCCTATTGCCACGGCGGAACTCATCGCCGCCTGCCTTGCCCGCTGCT
GGACAGGGGCTCGGCTGTTGGGCACTGACAATTCCGTGGTGTTGTCGGGGAAATCAT

CGTCCTTTCCTTGGCTGCTCGCCTGTGTTGCCACCTGGATTCTGCGCGGGACGTCCTT
CTGCTACGTCCCTTCGGCCCTCAATCCAGCGGACCTTCCTTCCCGCGGCCTGCTGCCG
GCTCTGCGGCCTCTTCCGCGTCTTCGCCTTCGCCCTCAGACGAGTCGGATCTCCCTTT
GGGCCGCCTCCCCGCGTCGACTTTAAGACCAATGACTTACAAGGCAGCTGTAGATCTT
AGCCACTTTTTAAAAGAAAAGGGGGGACTGGAAGGGCTAATTCACTCCCAACGAAG
ACAAGATCTGCTTTTTGCTTGTACTGGGTCTCTCTGGTTAGACCAGATCTGAGCCTGG
GAGCTCTCTGGCTAACTAGGGAACCCACTGCTTAAGCCTCAATAAAGCTTGCCTTGA
GTGCTTCAAGTAGTGTGTGCCCGTCTGTTGTGTGACTCTGGTAACTAGAGATCCCTCA
GACCCTTTTAGTCAGTGTGGAAAATCTCTAGCAGggcccgtttaaacccgctgatcagcctcgactgtgcctt
ctagttgccagccatctgttgtttgcccctcccccgtgccttccttgaccctggaaggtgccactcccactgtcctttcctaataaaatgaggaa
attgcatcgcattgtctgagtaggtgtcattctattctggggggtggggtggggcaggacagcaagggggaggattgggaagacaatagca
ggcatgctggggatgcggtgggctctatggcttctgaggcggaaagaaccagctggggctctagggggtatccccacgcgccctgtagc
ggcgcattaagcgcggcgggtgtggtggttacgcgcagcgtgaccgctacacttgccagcgccctagcgcccgctcctttcgctttcttccc
ttcctttctcgccacgttcgccggctttccccgtcaagctctaaatcgggggctccctttagggttccgatttagtgctttacggcacctcgaccc
caaaaaacttgattagggtgatggttcacgtagtgggccatcgccctgatagacggtttttcgccctttgacgttggagtccacgttctttaata
gtggactcttgttccaaactggaacaacactcaaccctatctcggtctattcttttgatttataatggttacaaataaagcaatagcatcacaaattt
cacaaataaagcatttttttcactgcattctagttgtggtttgtccaaactcatcaatgtatcttatcatgtctgtataccgtcgacctctagctagag
cttggcgtaatcatggtcatagctgtttcctgtgtgaaattgttatccgctcacaattccacacaacatacgagccggaagcataaagtgtaaag
cctggggtgcctaatgagtgagctaactcacattaattgcgttgcgctcactgcccgctttccagtcgggaaacctgtcgtgccagctgcatta
atgaatcggccaacgcgcggggagaggcggtttgcgtattgggcgctcttccgcttcctcgctcactgactcgctgcgctcggtcgttcggc
tgcggcgagcggtatcagctcactcaaaggcggtaatacggttatccacagaatcaggggataacgcaggaaagaacatgtgagcaaaa
ggccagcaaaaggccaggaaccgtaaaaaggccgcgttgctggcgtttttccataggctccgcccccctgacgagcatcacaaaaatcga
cgctcaagtcagaggtggcgaaacccgacaggactataaagataccaggcgtttccccctggaagctccctcgtgcgctctcctgttccga
ccctgccgcttaccggatacctgtccgcctttctcccttcgggaagcgtggcgctttctcatagctcacgctgtaggtatctcagttcggtgtag
gtcgttcgctccaagctgggctgtgtgcacgaaccccccgttcagcccgaccgctgcgccttatccggtaactatcgtcttgagtccaaccc
ggtaagacacgacttatcgccactggcagcagccactggtaacaggattagcagagcgaggtatgtaggcggtgctacagagttcttgaa
gtggtggcctaactacggctacactagaagaacagtatttggtatctgcgctctgctgaagccagttaccttcggaaaaagagttggtagctct
tgatccggcaaacaaaccaccgctggtagcggtggtttttttgtttgcaagcagcagattacgcgcagaaaaaaaggatctcaagaagatcc
tttgatcttttctacggggtctgacgctcagtggaacgaaaactcacgttaagggattttggtcatgagattatcaaaaaggatcttcacctagat
ccttttaaattaaaaatgaagttttaaatcaatctaaagtatatatgagtaaacttggtctgacagttaccaatgcttaatcagtgaggcacctatct
cagcgatctgtctatttcgttcatccatagttgcctgactccccgtcgtgtagataactacgatacgggagggcttaccatctggccccagtgct
gcaatgataccgcgagacccacgctcaccggctccagatttatcagcaataaaccagccagccggaagggccgagcgcagaagtggtc
ctgcaactttatccgcctccatccagtctattaattgttgccgggaagctagagtaagtagttcgccagttaatagtttgcgcaacgttgttgcca
ttgctacaggcatcgtggtgtcacgctcgtcgtttggtatggcttcattcagctccggttcccaacgatcaaggcgagttacatgatcccccat
gttgtgcaaaaaagcggttagctccttcggtcctccgatcgttgtcagaagtaagttggccgcagtgttatcactcatggttatggcagcactg
cataattctcttactgtcatgccatccgtaagatgcttttctgtgactggtgagtactcaaccaagtcattctgagaatagtgtatgcggcgaccg
agttgctcttgcccggcgtcaatacgggataataccgcgccacatagcagaactttaaaagtgctcatcattggaaaacgttcttcggggcga
aaactctcaaggatcttaccgctgttgagatccagttcgatgtaacccactcgtgcacccaactgatcttcagcatcttttactttcaccagcgttt
ctgggtgagcaaaaacaggaaggcaaaatgccgcaaaaaagggaataagggcgacacggaaatgttgaatactcatactcttcctttttca
atattattgaagcatttatcagggttattgtctcatgagcggatacatatttgaatgtatttagaaaaataaacaaataggggttccgcgcacattt

ccccgaaaagtgccacctgacgtcgacggatcgggagatctcccgatcccctatggtgcactctcagtacaatctgctctgatgccgcatag
ttaagccagtatctgctccctgcttgtgtgttggaggtcgctgagtagtgcgcgagcaaaatttaagctacaacaaggcaaggcttgaccgac
aattgcatgaagaatctgcttagggttaggcgttttgcgctgcttcgcgatgtacgggccagatatacgcgctgacattgattattga

4. **pLV-U6-[empty]-EFSNC-dSaCas9-KRAB-MeCp2-P2A-Puro-WPRE (pCL111) (no gRNA vector)**

gtcgacggatcgggagatctcccgatcccctatggtgcactctcagtacaatctgctctgatgccgcatagttaagccagtatctgctccctgc
ttgtgtgttggaggtcgctgagtagtgcgcgagcaaaatttaagctacaacaaggcaaggcttgaccgacaattgcatgaagaatctgcttag
ggttaggcgttttgcgctgcttcgcgatgtacgggccagatatacgcgttgacattgattattgactagttattaatagtaatcaattacggggtc
attagttcatagcccatatatggagttccgcgttacataacttacggtaaatggcccgcctggctgaccgcccaacgacccccgcccattgac
gtcaataatgacgtatgttcccatagtaacgccaatagggactttccattgacgtcaatgggtggagtatttacggtaaactgcccacttggca
gtacatcaagtgtatcatatgccaagtacgccccctattgacgtcaatgacggtaaatggcccgcctggcattatgcccagtacatgaccttat
gggactttcctacttggcagtacatctacgtattagtcatcgctattaccatggtgatgcggttttggcagtacatcaatgggcgtggatagcgg
tttgactcacggggatttccaagtctccaccccattgacgtcaatgggagtttgttttggcaccaaaatcaacgggactttccaaaatgtcgtaa
caactccgccccattgacgcaaatgggcggtaggcgtgtacggtgggaggtctatataagcagcgcgttttgcctgtactgggtctctctggt
tagaccagatctgagcctgggagctctctggctaactagggaacccactgcttaagcctcaataaagcttgccttgagtgcttcaagtagtgt
gtgcccgtctgttgtgtgactctggtaactagagatccctcagacccttttagtcagtgtggaaaatctctagcagtggcgcccgaacaggga
cttgaaagcgaaagggaaaccagaggagctctctcgacgcaggactcggcttgctgaagcgcgcacggcaagaggcgaggggcggc
gactggtgagtacgccaaaaattttgactagcggaggctagaaggagagagatgggtgcgagagcgtcagtattaagcgggggagaatt
agatcgcgatgggaaaaaattcggttaaggccagggggaaagaaaaaatataaattaaaacatatagtatgggcaagcagggagctagaa
cgattcgcagttaatcctggcctgttagaaacatcagaaggctgtagacaaatactgggacagctacaaccatcccttcagacaggatcaga
agaacttagatcattatataatacagtagcaaccctctattgtgtgcatcaaaggatagagataaaagacaccaaggaagctttagacaagat
agaggaagagcaaaacaaaagtaagaccaccgcacagcaagcggccgctgatcttcagacctggaggaggagatatgagggacaattg
gagaagtgaattatataaatataaagtagtaaaaattgaaccattaggagtagcacccaccaaggcaaagagaagagtggtgcagagaga
aaaaagagcagtgggaataggagctttgttccttgggttcttgggagcagcaggaagcactatgggcgcagcgtcaatgacgctgacggt
acaggccagacaattattgtctggtatagtgcagcagcagaacaatttgctgagggctattgaggcgcaacagcatctgttgcaactcacagt
ctggggcatcaagcagctccaggcaagaatcctggctgtggaaagatacctaaaggatcaacagctcctggggatttggggttgctctgga
aaactcatttgcaccactgctgtgccttggaatgctagttggagtaataaatctctggaacagatttggaatcacacgacctggatggagtggg
acagagaaattaacaattacacaagcttaatacactccttaattgaagaatcgcaaaaccagcaagaaaagaatgaacaagaattattggaat
tagataaatgggcaagtttgtggaattggtttaacataacaaattggctgtggtatataaaattattcataatgatagtaggaggcttggtaggttt
aagaatagtttttgctgtactttctatagtgaatagagttaggcagggatattcaccattatcgtttcagacccacctcccaaccccgaggggac
ccgacaggcccgaaggaatagaagaagaaggtggagagagagacagagacagatccattcgattagtgaacggatcggcactgcgtgc
gccaattctgcagacaaatggcagtattcatccacaattttaaaagaaaaggggggattggggggtacagtgcaggggaaagaatagtaga
cataatagcaacagacatacaaactaaagaattacaaaaacaaattacaaaaattcaaaattttcgggtttattacagggacagcagagatcc
agtttggTTAATTAATGGGCGGGACGTTAACGGGGCGGAACGGTACCgagggcctatttcccatgattc
cttcatatttgcatatacgatacaaggctgttagagagataattagaattaatttgactgtaaacacaaagatattagtacaaaatacgtgacgta
gaaagtaataatttcttgggtagtttgcagttttaaaattatgttttaaaatggactatcatatgcttaccgtaacttgaaagtatttcgatttcttggct
ttatatatcttGTGGAAAGGACGAAAcaccggagacgtgtacacgtctctgTTTtagtactctggaaacagaatctactaaaa
caaggcaaaatgccgtgtttatctcgtcaacttgttggcgagaTTTTTTgaattcgctagctaggtcttgaaaggagtgggaattggctcc

ggtgcccgtcagtgggcagagcgcacatcgcccacagtccccgagaagttggggggaggggtcggcaattgatccggtgcctagagaa
ggtggcgcggggtaaactgggaaagtgatgtcgtgtactggctccgcctttttcccgagggtgggggagaaccgtatataagtgcagtagt
cgccgtgaacgttctttttcgcaacgggtttgccgccagaacacaggaccggtgccaccatgGACTACAAAGACCATGAC
GGTGATTATAAAGATCATGACATaGATTACAAGGATGACGATGACAAGggtggatcgccaaaga
aaaagaggaaagtcgccccaaagaagaagcggaaggtcggtatccacggagtcccagcagccaagcggaactacatcctgggcctgg
ccatcggcatcaccagcgtgggctacggcatcatcgactacgagacacgggacgtgatcgatgccggcgtgcggctgttcaaagaggcc
aacgtggaaaacaacgagggcaggcggagcaagagaggcgccagaaggctgaagcggcggaggcggcatagaatccagagagtga
agaagctgctgttcgactacaacctgctgaccgaccacagcgagctgagcggcatcaacccctacgaggccagagtgaagggcctgagc
cagaagctgagcgaggaagagttctctgccgccctgctgcacctggccaagagaagaggcgtgcacaacgtgaacgaggtggaagag
gacaccggcaacgagctgtccaccaaagagcagatcagccggaacagcaaggccctggaagagaaatacgtggccgaactgcagctg
gaacggctgaagaaagacggcgaagtgcggggcagcatcaacagattcaagaccagcgactacgtgaaagaagccaaacagctgctg
aaggtgcagaaggcctaccaccagctggaccagagcttcatcgacacctacatcgacctgctggaaacccggcggacctactatgaggg
acctggcgagggcagccccttcggctggaaggacatcaaagaatggtacgagatgctgatgggccactgcacctacttccccgaggaact
gcggagcgtgaagtacgcctacaacgccgacctgtacaacgccctgaacgacctgaacaatctcgtgatcaccagggacgagaacgag
aagctggaatattacgagaagttccagatcatcgagaacgtgttcaagcagaagaagaagcccaccctgaagcagatcgccaaagaaatc
ctcgtgaacgaagaggatattaagggctacagagtgaccagcaccggcaagcccgagttcaccaacctgaaggtgtaccacgacatcaa
ggacattaccgcccggaaagagattattgagaacgccgagctgctggatcagattgccaagatcctgaccatctaccagagcagcgagga
catccaggaagaactgaccaatctgaactccgagctgacccaggaagagatcgagcagatctctaatctgaagggctataccggcaccca
caacctgagcctgaaggccatcaacctgatcctggacgagctgtggcacaccaacgacaaccagatcgctatcttcaaccggctgaagct
ggtgcccaagaaggtggacctgtcccagcagaaagagatccccaccaccctggtggacgacttcatcctgagccccgtcgtgaagagaa
gcttcatccagagcatcaaagtgatcaacgccatcatcaagaagtacggcctgcccaacgacatcattatcgagctggcccgcgagaaga
actccaaggacgcccagaaaatgatcaacgagatgcagaagcggaaccggcagaccaacgagcggatcgaggaaatcatccggacca
ccggcaaagagaacgccaagtacctgatcgagaagatcaagctgcacgacatgcaggaaggcaagtgcctgtacagcctggaagccat
ccctctggaagatctgctgaacaaccccttcaactatgaggtggaccacatcatccccagaagcgtgtccttcgacaacagcttcaacaaca
aggtgctcgtgaagcaggaagaagccagcaagaagggcaaccggaccccattccagtacctgagcagcagcgacagcaagatcagct
acgaaaccttcaagaagcacatcctgaatctggccaagggcaagggcagaatcagcaagaccaagaaagagtatctgctggaagaacg
ggacatcaacaggttctccgtgcagaaagacttcatcaaccggaacctggtggataccagatacgccaccagaggcctgatgaacctgct
gcggagctacttcagagtgaacaacctggacgtgaaagtgaagtccatcaatggcggcttcaccagctttctgcggcggaagtggaagttt
aagaaagagcggaacaaggggtacaagcaccacgccgaggacgccctgatcattgccaacgccgatttcatcttcaaagagtggaagaa
actggacaaggccaaaaaagtgatggaaaaccagatgttcgaggaaaagcaggccgagagcatgcccgagatcgaaaccgagcagga
gtacaaagagatcttcatcacccccccaccagatcaagcacattaaggacttcaaggactacaagtacagccaccgggtggacaagaagcc
taatagagagctgattaacgacaccctgtactccacccggaaggacgacaagggcaacaccctgatcgtgaacaatctgaacggcctgta
cgacaaggacaatgacaagctgaaaaagctgatcaacaagagccccgaaaagctgctgatgtaccaccacgacccccagacctaccag
aaactgaagctgattatggaacagtacggcgacgagaagaatcccctgtacaagtactacgaggaaaccgggaactacctgaccaagtac
tccaaaaaggacaacggccccgtgatcaagaagattaagtattacggcaacaaactgaacgcccatctggacatcaccgacgactacccc
aacagcagaaacaaggtcgtgaagctgtccctgaagccctacagattcgacgtgtacctggacaatggcgtgtacaagttcgtgaccgtga
agaatctggatgtgatcaaaaaagaaaactactacgaagtgaatagcaagtgctatgaggaagctaagaagctgaagaagatcagcaacc
aggccgagtttatcgcctccttctacaacaacgatctgatcaagatcaacggcgagctgtatagagtgatcggcgtgaacaacgacctgctg
aaccggatcgaagtgaacatgatcgacatcacctaccgcgagtacctggaaaacatgaacgacaagaggcccccccaggatcattaagac
aatcgcctccaagacccagagcattaagaagtacagcacagacattctgggcaacctgtatgaagtgaaatctaagaagcaccctcagatc

atcaaaaagggcaaaaggccggcggccacgaaaaaggcGcTCGAgggtggaggaagtggcgggtcagggtcgggtggcAGC
GCTtcagggtcgggtggcACTAGTcggacactggtgaccttcaaggatgtatttgtggacttcaccagggaggagtggaagctgct
ggacactgctcagcagatcgtgtacagaaatgtgatgctggagaactataagaacctggtttccttgggttatcagcttactaagccagatgtg
atcctccggttggagaagggagaagagccctcgggaggtggttcgggaggtggttcggagggtgtgcaggtgaaaagggtcctggaga
aaagtcctgggaagctccttgtcaagatgccttttcaaacttcgccaggggcaaggctgagggggtggggccaccacatccacccagg
tcatggtgatcaaacgccccggcaggaagcgaaaagctgaggccgaccctcaggccattcccaagaaacggggccgaaagccgggga
gtgtggtggcagccgctgccgccgaggccaaaaagaaagccgtgaaggagtcttctatccgatctgtgcaggagacAgtactccccatc
aagaagcgcaagacccgggagGCTAGCggaggtggttcGCCTAGGggaggtggttcgccaaagaagaaacggaaggtgg
gCCGGcccggatccGGCGCAACAAACTTCTCTCTGCTGAAACAAGCCGGAGATGTCGAAG
AGAATCCTGGACCGACCGAGTACAAGCCCACGGTGCGCCTCGCCACCCGCGACGAC
GTCCCCAGGGCCGTACGCACCCTCGCCGCCGCGTTCGCCGACTACCCCGCCACGCGC
CACACCGTCGATCCGGACCGCCACATCGAGCGGGTCACCGAGCTGCAAGAACTCTTC
CTCACGCGCGTCGGGCTCGACATCGGCAAGGTGTGGGTCGCGGACGACGGCGCCGC
GGTGGCGGTCTGGACCACGCCGGAGAGCGTCGAAGCGGGGGCGGTGTTCGCCGAGA
TCGGCCCGCGCATGGCCGAGTTGAGCGGTTCCCGGCTGGCCGCGCAGCAACAGATG
GAAGGCCTCCTGGCGCCGCACCGGCCCAAGGAGCCCGCGTGGTTCCTGGCCACCGT
CGGAGTCTCGCCCGACCACCAGGGCAAGGGTCTGGGCAGCGCCGTCGTGCTCCCCG
GAGTGGAGGCGGCCGAGCGCGCCGGGGTGCCCGCCTTCCTGGAGACCTCCGCGCCC
CGCAACCTCCCCTTCTACGAGCGGCTCGGCTTCACCGTCACCGCCGACGTCGAGGTG
CCCGAAGGACCGCGCACCTGGTGCATGACCCGCAAGCCCGGTGCCTGAACGCGTTA
AGTCGACAATCAACCTCTGGATTACAAAATTTGTGAAAGATTGACTGGTATTCTTAAC
TATGTTGCTCCTTTTACGCTATGTGGATACGCTGCTTTAATGCCTTTGTATCATGCTATT
GCTTCCCGTATGGCTTTCATTTTCTCCTCCTTGTATAAATCCTGGTTGCTGTCTCTTTAT
GAGGAGTTGTGGCCCGTTGTCAGGCAACGTGGCGTGGTGTGCACTGTGTTTGCTGAC
GCAACCCCCACTGGTTGGGGCATTGCCACCACCTGTCAGCTCCTTTCCGGGACTTTC
GCTTTCCCCCTCCCTATTGCCACGGCGGAACTCATCGCCGCCTGCCTTGCCCGCTGCT
GGACAGGGGCTCGGCTGTTGGGCACTGACAATTCCGTGGTGTTGTCGGGGAAATCAT
CGTCCTTTCCTTGGCTGCTCGCCTGTGTTGCCACCTGGATTCTGCGCGGGACGTCCTT
CTGCTACGTCCCTTCGGCCCTCAATCCAGCGGACCTTCCTTCCCGCGGCCTGCTGCCG
GCTCTGCGGCCTCTTCCGCGTCTTCGCCTTCGCCCTCAGACGAGTCGGATCTCCCTTT
GGGCCGCCTCCCCGCGTCGACTTTAAGACCAATGACTTACAAGGCAGCTGTAGATCTT
AGCCACTTTTTAAAAGAAAAGGGGGGACTGGAAGGGCTAATTCACTCCCAACGAAG
ACAAGATCTGCTTTTTGCTTGTACTGGGTCTCTCTGGTTAGACCAGATCTGAGCCTGG
GAGCTCTCTGGCTAACTAGGGAACCCACTGCTTAAGCCTCAATAAAGCTTGCCTTGA
GTGCTTCAAGTAGTGTGTGCCCGTCTGTTGTGTGACTCTGGTAACTAGAGATCCCTCA
GACCCTTTTAGTCAGTGTGGAAAATCTCTAGCAGggcccgtttaaacccgctgatcagcctcgactgtgcctt
ctagttgccagccatctgttgtttgcccctcccccgtgccttccttgaccctggaaggtgccactcccactgtcctttcctaataaaatgaggaa
attgcatcgcattgtctgagtaggtgtcattctattctggggggtggggtggggcaggacagcaagggggaggattgggaagacaatagca
ggcatgctggggatgcggtgggctctatggcttctgaggcggaaagaaccagctggggctctagggggtatccccacgcgccctgtagc
ggcgcattaagcgcggcgggtgtggtggttacgcgcagcgtgaccgctacacttgccagcgccctagcgcccgctcctttcgctttcttccc

ttcctttctcgccacgttcgccggctttccccgtcaagctctaaatcgggggctccctttagggttccgatttagtgctttacggcacctcgaccc
caaaaaacttgattagggtgatggttcacgtagtgggccatcgccctgatagacggtttttcgccctttgacgttggagtccacgttctttaata
gtggactcttgttccaaactggaacaacactcaaccctatctcggtctattcttttgatttataatggttacaaataaagcaatagcatcacaaattt
cacaaataaagcatttttttcactgcattctagttgtggtttgtccaaactcatcaatgtatcttatcatgtctgtataccgtcgacctctagctagag
cttggcgtaatcatggtcatagctgtttcctgtgtgaaattgttatccgctcacaattccacacaacatacgagccggaagcataaagtgtaaag
cctggggtgcctaatgagtgagctaactcacattaattgcgttgcgctcactgcccgctttccagtcgggaaacctgtcgtgccagctgcatta
atgaatcggccaacgcgcggggagaggcggtttgcgtattgggcgctcttccgcttcctcgctcactgactcgctgcgctcggtcgttcggc
tgcggcgagcggtatcagctcactcaaaggcggtaatacggttatccacagaatcaggggataacgcaggaaagaacatgtgagcaaaa
ggccagcaaaaggccaggaaccgtaaaaaggccgcgttgctggcgtttttccataggctccgcccccctgacgagcatcacaaaaatcga
cgctcaagtcagaggtggcgaaacccgacaggactataaagataccaggcgtttccccctggaagctccctcgtgcgctctcctgttccga
ccctgccgcttaccggatacctgtccgcctttctcccttcgggaagcgtggcgctttctcatagctcacgctgtaggtatctcagttcggtgtag
gtcgttcgctccaagctgggctgtgtgcacgaaccccccgttcagcccgaccgctgcgccttatccggtaactatcgtcttgagtccaaccc
ggtaagacacgacttatcgccactggcagcagccactggtaacaggattagcagagcgaggtatgtaggcggtgctacagagttcttgaa
gtggtggcctaactacggctacactagaagaacagtatttggtatctgcgctctgctgaagccagttaccttcggaaaaagagttggtagctct
tgatccggcaaacaaaccaccgctggtagcggtggtttttttgtttgcaagcagcagattacgcgcagaaaaaaaggatctcaagaagatcc
tttgatcttttctacggggtctgacgctcagtggaacgaaaactcacgttaagggattttggtcatgagattatcaaaaaggatcttcacctagat
ccttttaaattaaaaatgaagttttaaatcaatctaaagtatatatgagtaaacttggtctgacagttaccaatgcttaatcagtgaggcacctatct
cagcgatctgtctatttcgttcatccatagttgcctgactccccgtcgtgtagataactacgatacgggagggcttaccatctggccccagtgct
gcaatgataccgcgagacccacgctcaccggctccagatttatcagcaataaaccagccagccggaagggccgagcgcagaagtggtc
ctgcaactttatccgcctccatccagtctattaattgttgccgggaagctagagtaagtagttcgccagttaatagtttgcgcaacgttgttgcca
ttgctacaggcatcgtggtgtcacgctcgtcgtttggtatggcttcattcagctccggttcccaacgatcaaggcgagttacatgatcccccat
gttgtgcaaaaaagcggttagctccttcggtcctccgatcgttgtcagaagtaagttggccgcagtgttatcactcatggttatggcagcactg
cataattctcttactgtcatgccatccgtaagatgcttttctgtgactggtgagtactcaaccaagtcattctgagaatagtgtatgcggcgaccg
agttgctcttgcccggcgtcaatacgggataataccgcgccacatagcagaactttaaaagtgctcatcattggaaaacgttcttcggggcga
aaactctcaaggatcttaccgctgttgagatccagttcgatgtaacccactcgtgcacccaactgatcttcagcatcttttactttcaccagcgttt
ctgggtgagcaaaaacaggaaggcaaaatgccgcaaaaaagggaataagggcgacacggaaatgttgaatactcatactcttcctttttca
atattattgaagcatttatcagggttattgtctcatgagcggatacatatttgaatgtatttagaaaaataaacaaataggggttccgcgcacattt
ccccgaaaagtgccacctgac

5. pBK1841- pLV-U6-[SNCAintron1 gRNA 1]-EFSNC-dSaCas9-KRAB-MeCp2-P2A-Puro-WPRE (THERAPEUTIC VECTOR)

gtcgacggatcgggagatctcccgatcccctatggtgcactctcagtacaatctgctctgatgccgcatagttaagccagtatctgctccctgc
ttgtgtgttggaggtcgctgagtagtgcgcgagcaaaatttaagctacaacaaggcaaggcttgaccgacaattgcatgaagaatctgcttag
ggttaggcgttttgcgctgcttcgcgatgtacgggccagatatacgcgttgacattgattattgactagttattaatagtaatcaattacggggtc
attagttcatagcccatatatggagttccgcgttacataacttacggtaaatggcccgcctggctgaccgcccaacgacccccgcccattgac
gtcaataatgacgtatgttcccatagtaacgccaatagggactttccattgacgtcaatgggtggagtatttacggtaaactgcccacttggca
gtacatcaagtgtatcatatgccaagtacgccccctattgacgtcaatgacggtaaatggcccgcctggcattatgcccagtacatgaccttat
gggactttcctacttggcagtacatctacgtattagtcatcgctattaccatggtgatgcggttttggcagtacatcaatgggcgtggatagcgg
tttgactcacggggatttccaagtctccaccccattgacgtcaatgggagtttgttttggcaccaaaatcaacgggactttccaaaatgtcgtaa
caactccgccccattgacgcaaatgggcggtaggcgtgtacggtgggaggtctatataagcagcgcgttttgcctgtactgggtctctctggt

tagaccagatctgagcctgggagctctctggctaactagggaacccactgcttaagcctcaataaagcttgccttgagtgcttcaagtagtgt
gtgcccgtctgttgtgtgactctggtaactagagatccctcagacccttttagtcagtgtggaaaatctctagcagtggcgcccgaacaggga
cttgaaagcgaaagggaaaccagaggagctctctcgacgcaggactcggcttgctgaagcgcgcacggcaagaggcgaggggcggc
gactggtgagtacgccaaaaattttgactagcggaggctagaaggagagagatgggtgcgagagcgtcagtattaagcgggggagaatt
agatcgcgatgggaaaaaattcggttaaggccagggggaaagaaaaaatataaattaaaacatatagtatgggcaagcagggagctagaa
cgattcgcagttaatcctggcctgttagaaacatcagaaggctgtagacaaatactgggacagctacaaccatcccttcagacaggatcaga
agaacttagatcattatataatacagtagcaaccctctattgtgtgcatcaaaggatagagataaaagacaccaaggaagctttagacaagat
agaggaagagcaaaacaaaagtaagaccaccgcacagcaagcggccgctgatcttcagacctggaggaggagatatgagggacaattg
gagaagtgaattatataaatataaagtagtaaaaattgaaccattaggagtagcacccaccaaggcaaagagaagagtggtgcagagaga
aaaaagagcagtgggaataggagctttgttccttgggttcttgggagcagcaggaagcactatgggcgcagcgtcaatgacgctgacggt
acaggccagacaattattgtctggtatagtgcagcagcagaacaatttgctgagggctattgaggcgcaacagcatctgttgcaactcacagt
ctggggcatcaagcagctccaggcaagaatcctggctgtggaaagatacctaaaggatcaacagctcctggggatttggggttgctctgga
aaactcatttgcaccactgctgtgccttggaatgctagttggagtaataaatctctggaacagatttggaatcacacgacctggatggagtggg
acagagaaattaacaattacacaagcttaatacactccttaattgaagaatcgcaaaaccagcaagaaaagaatgaacaagaattattggaat
tagataaatgggcaagtttgtggaattggtttaacataacaaattggctgtggtatataaaattattcataatgatagtaggaggcttggtaggttt
aagaatagtttttgctgtactttctatagtgaatagagttaggcagggatattcaccattatcgtttcagacccacctcccaaccccgaggggac
ccgacaggcccgaaggaatagaagaagaaggtggagagagagacagagacagatccattcgattagtgaacggatcggcactgcgtgc
gccaattctgcagacaaatggcagtattcatccacaattttaaaagaaaaggggggattggggggtacagtgcaggggaaagaatagtaga
cataatagcaacagacatacaaactaaagaattacaaaaacaaattacaaaaattcaaaattttcgggtttattacagggacagcagagatcc
agtttggTTAATTAATGGGCGGGACGTTAACGGGGCGGAACGGTACCgagggcctatttcccatgattc
cttcatatttgcatatacgatacaaggctgttagagagataattagaattaatttgactgtaaacacaaagatattagtacaaaatacgtgacgta
gaaagtaataatttcttgggtagtttgcagttttaaaattatgttttaaaatggactatcatatgcttaccgtaacttgaaagtatttcgatttcttggct
ttatatatcttGTGGAAAGGACGAAAcaccgACCTCCCAGAGACCTGGCCCAgTTTtagtactctggaa
acagaatctactaaaacaaggcaaaatgccgtgtttatctcgtcaacttgttggcgagaTTTTTTgaattcgctagctaggtcttgaaagg
agtgggaattggctccggtgcccgtcagtgggcagagcgcacatcgcccacagtccccgagaagttggggggaggggtcggcaattgat
ccggtgcctagagaaggtggcgcggggtaaactgggaaagtgatgtcgtgtactggctccgcctttttcccgagggtgggggagaaccgt
atataagtgcagtagtcgccgtgaacgttctttttcgcaacgggtttgccgccagaacacaggaccggtgccaccatgGACTACAA
AGACCATGACGGTGATTATAAAGATCATGACATaGATTACAAGGATGACGATGACAAG
ggtggatcgccaaagaaaaagaggaaagtcgccccaaagaagaagcggaaggtcggtatccacggagtcccagcagccaagcggaa
ctacatcctgggcctggccatcggcatcaccagcgtgggctacggcatcatcgactacgagacacgggacgtgatcgatgccggcgtgc
ggctgttcaaagaggccaacgtggaaaacaacgagggcaggcggagcaagagaggcgccagaaggctgaagcggcggaggcggca
tagaatccagagagtgaagaagctgctgttcgactacaacctgctgaccgaccacagcgagctgagcggcatcaacccctacgaggcca
gagtgaagggcctgagccagaagctgagcgaggaagagttctctgccgccctgctgcacctggccaagagaagaggcgtgcacaacgt
gaacgaggtggaagaggacaccggcaacgagctgtccaccaaagagcagatcagccggaacagcaaggccctggaagagaaatacg
tggccgaactgcagctggaacggctgaagaaagacggcgaagtgcggggcagcatcaacagattcaagaccagcgactacgtgaaag
aagccaaacagctgctgaaggtgcagaaggcctaccaccagctggaccagagcttcatcgacacctacatcgacctgctggaaacccgg
cggacctactatgagggacctggcgagggcagccccttcggctggaaggacatcaaagaatggtacgagatgctgatgggccactgcac
ctacttccccgaggaactgcggagcgtgaagtacgcctacaacgccgacctgtacaacgccctgaacgacctgaacaatctcgtgatcac
cagggacgagaacgagaagctggaatattacgagaagttccagatcatcgagaacgtgttcaagcagaagaagaagcccaccctgaagc
agatcgccaaagaaatcctcgtgaacgaagaggatattaagggctacagagtgaccagcaccggcaagcccgagttcaccaacctgaag

gtgtaccacgacatcaaggacattaccgcccggaaagagattattgagaacgccgagctgctggatcagattgccaagatcctgaccatct
accagagcagcgaggacatccaggaagaactgaccaatctgaactccgagctgacccaggaagagatcgagcagatctctaatctgaag
ggctataccggcacccacaacctgagcctgaaggccatcaacctgatcctggacgagctgtggcacaccaacgacaaccagatcgctatc
ttcaaccggctgaagctggtgcccaagaaggtggacctgtcccagcagaaagagatccccaccaccctggtggacgacttcatcctgagc
cccgtcgtgaagagaagcttcatccagagcatcaaagtgatcaacgccatcatcaagaagtacggcctgcccaacgacatcattatcgagc
tggcccgcgagaagaactccaaggacgcccagaaaatgatcaacgagatgcagaagcggaaccggcagaccaacgagcggatcgag
gaaatcatccggaccaccggcaaagagaacgccaagtacctgatcgagaagatcaagctgcacgacatgcaggaaggcaagtgcctgt
acagcctggaagccatccctctggaagatctgctgaacaaccccttcaactatgaggtggaccacatcatccccagaagcgtgtccttcgac
aacagcttcaacaacaaggtgctcgtgaagcaggaagaagccagcaagaagggcaaccggaccccattccagtacctgagcagcagcg
acagcaagatcagctacgaaaccttcaagaagcacatcctgaatctggccaagggcaagggcagaatcagcaagaccaagaaagagtat
ctgctggaagaacgggacatcaacaggttctccgtgcagaaagacttcatcaaccggaacctggtggataccagatacgccaccagaggc
ctgatgaacctgctgcggagctacttcagagtgaacaacctggacgtgaaagtgaagtccatcaatggcggcttcaccagctttctgcggcg
gaagtggaagtttaagaaagagcggaacaaggggtacaagcaccacgccgaggacgccctgatcattgccaacgccgatttcatcttcaa
agagtggaagaaactggacaaggccaaaaaagtgatggaaaaccagatgttcgaggaaaagcaggccgagagcatgcccgagatcga
aaccgagcaggagtacaaagagatcttcatcacccccccaccagatcaagcacattaaggacttcaaggactacaagtacagccaccgggt
ggacaagaagcctaatagagagctgattaacgacaccctgtactccacccggaaggacgacaagggcaacaccctgatcgtgaacaatct
gaacggcctgtacgacaaggacaatgacaagctgaaaaagctgatcaacaagagccccgaaaagctgctgatgtaccaccacgaccccc
agacctaccagaaactgaagctgattatggaacagtacggcgacgagaagaatcccctgtacaagtactacgaggaaaccgggaactac
ctgaccaagtactccaaaaaggacaacggccccgtgatcaagaagattaagtattacggcaacaaactgaacgcccatctggacatcacc
gacgactaccccaacagcagaaacaaggtcgtgaagctgtccctgaagccctacagattcgacgtgtacctggacaatggcgtgtacaag
ttcgtgaccgtgaagaatctggatgtgatcaaaaaagaaaactactacgaagtgaatagcaagtgctatgaggaagctaagaagctgaaga
agatcagcaaccaggccgagtttatcgcctccttctacaacaacgatctgatcaagatcaacggcgagctgtatagagtgatcggcgtgaac
aacgacctgctgaaccggatcgaagtgaacatgatcgacatcacctaccgcgagtacctggaaaacatgaacgacaagaggcccccccag
gatcattaagacaatcgcctccaagacccagagcattaagaagtacagcacagacattctgggcaacctgtatgaagtgaaatctaagaag
caccctcagatcatcaaaaagggcaaaaggccggcggccacgaaaaaggcGcTCGAgggtggaggaagtggcgggtcagggtc
gggtggcAGCGCTtcagggtcgggtggcACTAGTcggacactggtgaccttcaaggatgtatttgtggacttcaccagggagga
gtggaagctgctggacactgctcagcagatcgtgtacagaaatgtgatgctggagaactataagaacctggtttccttgggttatcagcttact
aagccagatgtgatcctccggttggagaagggagaagagccctcgggaggtggttcgggaggtggttcggagggtgtgcaggtgaaaa
gggtcctggagaaaagtcctgggaagctccttgtcaagatgccttttcaaacttcgccagggggcaaggctgaggggggtggggccacca
catccacccaggtcatggtgatcaaacgccccggcaggaagcgaaaagctgaggccgaccctcaggccattcccaagaaacggggccg
aaagccggggagtgtggtggcagccgctgccgccgaggccaaaaagaaagccgtgaaggagtcttctatccgatctgtgcaggagacA
gtactccccatcaagaagcgcaagacccgggagGCTAGCggaggtggttcGCCTAGGggaggtggttcgccaaagaagaaa
cggaaggtgggCCGGcccggatccGGCGCAACAAACTTCTCTCTGCTGAAACAAGCCGGAGAT
GTCGAAGAGAATCCTGGACCGACCGAGTACAAGCCCACGGTGCGCCTCGCCACCCG
CGACGACGTCCCCAGGGCCGTACGCACCCTCGCCGCCGCGTTCGCCGACTACCCCGC
CACGCGCCACACCGTCGATCCGGACCGCCACATCGAGCGGGTCACCGAGCTGCAAG
AACTCTTCCTCACGCGCGTCGGGCTCGACATCGGCAAGGTGTGGGTCGCGGACGACG
GCGCCGCGGTGGCGGTCTGGACCACGCCGGAGAGCGTCGAAGCGGGGGCGGTGTTC
GCCGAGATCGGCCCGCGCATGGCCGAGTTGAGCGGTTCCCGGCTGGCCGCGCAGCA
ACAGATGGAAGGCCTCCTGGCGCCGCACCGGCCCAAGGAGCCCGCGTGGTTCCTGG

CCACCGTCGGAGTCTCGCCCGACCACCAGGGCAAGGGTCTGGGCAGCGCCGTCGTG
CTCCCCGGAGTGGAGGCGGCCGAGCGCGCCGGGGTGCCCGCCTTCCTGGAGACCTC
CGCGCCCCGCAACCTCCCCTTCTACGAGCGGCTCGGCTTCACCGTCACCGCCGACGT
CGAGGTGCCCGAAGGACCGCGCACCTGGTGCATGACCCGCAAGCCCGGTGCCTGAA
CGCGTTAAGTCGACAATCAACCTCTGGATTACAAAATTTGTGAAAGATTGACTGGTAT
TCTTAACTATGTTGCTCCTTTTACGCTATGTGGATACGCTGCTTTAATGCCTTTGTATCA
TGCTATTGCTTCCCGTATGGCTTTCATTTTCTCCTCCTTGTATAAATCCTGGTTGCTGTC
TCTTTATGAGGAGTTGTGGCCCGTTGTCAGGCAACGTGGCGTGGTGTGCACTGTGTTT
GCTGACGCAACCCCCACTGGTTGGGGCATTGCCACCACCTGTCAGCTCCTTTCCGGG
ACTTTCGCTTTCCCCCTCCCTATTGCCACGGCGGAACTCATCGCCGCCTGCCTTGCCC
GCTGCTGGACAGGGGCTCGGCTGTTGGGCACTGACAATTCCGTGGTGTTGTCGGGGA
AATCATCGTCCTTTCCTTGGCTGCTCGCCTGTGTTGCCACCTGGATTCTGCGCGGGAC
GTCCTTCTGCTACGTCCCTTCGGCCCTCAATCCAGCGGACCTTCCTTCCCGCGGCCTG
CTGCCGGCTCTGCGGCCTCTTCCGCGTCTTCGCCTTCGCCCTCAGACGAGTCGGATCT
CCCTTTGGGCCGCCTCCCCGCGTCGACTTTAAGACCAATGACTTACAAGGCAGCTGTA
GATCTTAGCCACTTTTTAAAAGAAAAGGGGGGACTGGAAGGGCTAATTCACTCCCAA
CGAAGACAAGATCTGCTTTTTGCTTGTACTGGGTCTCTCTGGTTAGACCAGATCTGAG
CCTGGGAGCTCTCTGGCTAACTAGGGAACCCACTGCTTAAGCCTCAATAAAGCTTGC
CTTGAGTGCTTCAAGTAGTGTGTGCCCGTCTGTTGTGTGACTCTGGTAACTAGAGATC
CCTCAGACCCTTTTAGTCAGTGTGGAAAATCTCTAGCAGggcccgtttaaacccgctgatcagcctcga
ctgtgccttctagttgccagccatctgttgtttgcccctcccccgtgccttccttgaccctggaaggtgccactcccactgtcctttcctaataaa
atgaggaaattgcatcgcattgtctgagtaggtgtcattctattctggggggtggggtggggcaggacagcaagggggaggattgggaag
acaatagcaggcatgctggggatgcggtgggctctatggcttctgaggcggaaagaaccagctggggctctagggggtatccccacgcg
ccctgtagcggcgcattaagcgcggcgggtgtggtggttacgcgcagcgtgaccgctacacttgccagcgccctagcgcccgctcctttc
gctttcttcccttcctttctcgccacgttcgccggctttccccgtcaagctctaaatcgggggctccctttagggttccgatttagtgctttacggc
acctcgaccccaaaaaacttgattagggtgatggttcacgtagtgggccatcgccctgatagacggtttttcgccctttgacgttggagtccac
gttctttaatagtggactcttgttccaaactggaacaacactcaaccctatctcggtctattcttttgatttataatggttacaaataaagcaatagc
atcacaaatttcacaaataaagcatttttttcactgcattctagttgtggtttgtccaaactcatcaatgtatcttatcatgtctgtataccgtcgacct
ctagctagagcttggcgtaatcatggtcatagctgtttcctgtgtgaaattgttatccgctcacaattccacacaacatacgagccggaagcata
aagtgtaaagcctggggtgcctaatgagtgagctaactcacattaattgcgttgcgctcactgcccgctttccagtcgggaaacctgtcgtgc
cagctgcattaatgaatcggccaacgcgcggggagaggcggtttgcgtattgggcgctcttccgcttcctcgctcactgactcgctgcgctc
ggtcgttcggctgcggcgagcggtatcagctcactcaaaggcggtaatacggttatccacagaatcaggggataacgcaggaaagaacat
gtgagcaaaaggccagcaaaaggccaggaaccgtaaaaaggccgcgttgctggcgtttttccataggctccgcccccctgacgagcatc
acaaaaatcgacgctcaagtcagaggtggcgaaacccgacaggactataaagataccaggcgtttccccctggaagctccctcgtgcgct
ctcctgttccgaccctgccgcttaccggatacctgtccgcctttctcccttcgggaagcgtggcgctttctcatagctcacgctgtaggtatctc
agttcggtgtaggtcgttcgctccaagctgggctgtgtgcacgaaccccccgttcagcccgaccgctgcgccttatccggtaactatcgtctt
gagtccaacccggtaagacacgacttatcgccactggcagcagccactggtaacaggattagcagagcgaggtatgtaggcggtgctaca
gagttcttgaagtggtggcctaactacggctacactagaagaacagtatttggtatctgcgctctgctgaagccagttaccttcggaaaaaga
gttggtagctcttgatccggcaaacaaaccaccgctggtagcggtggtttttttgtttgcaagcagcagattacgcgcagaaaaaaaggatct
caagaagatcctttgatcttttctacggggtctgacgctcagtggaacgaaaactcacgttaagggattttggtcatgagattatcaaaaaggat

cttcacctagatccttttaaattaaaaatgaagttttaaatcaatctaaagtatatatgagtaaacttggtctgacagttaccaatgcttaatcagtg
aggcacctatctcagcgatctgtctatttcgttcatccatagttgcctgactccccgtcgtgtagataactacgatacgggagggcttaccatct
ggccccagtgctgcaatgataccgcgagacccacgctcaccggctccagatttatcagcaataaaccagccagccggaagggccgagc
gcagaagtggtcctgcaactttatccgcctccatccagtctattaattgttgccgggaagctagagtaagtagttcgccagttaatagtttgcgc
aacgttgttgccattgctacaggcatcgtggtgtcacgctcgtcgtttggtatggcttcattcagctccggttcccaacgatcaaggcgagttac
atgatcccccatgttgtgcaaaaaagcggttagctccttcggtcctccgatcgttgtcagaagtaagttggccgcagtgttatcactcatggtta
tggcagcactgcataattctcttactgtcatgccatccgtaagatgcttttctgtgactggtgagtactcaaccaagtcattctgagaatagtgtat
gcggcgaccgagttgctcttgcccggcgtcaatacgggataataccgcgccacatagcagaactttaaaagtgctcatcattggaaaacgtt
cttcggggcgaaaactctcaaggatcttaccgctgttgagatccagttcgatgtaacccactcgtgcacccaactgatcttcagcatcttttactt
tcaccagcgtttctgggtgagcaaaaacaggaaggcaaaatgccgcaaaaaagggaataagggcgacacggaaatgttgaatactcatac
tcttcctttttcaatattattgaagcatttatcagggttattgtctcatgagcggatacatatttgaatgtatttagaaaaataaacaaataggggttc
cgcgcacatttccccgaaaagtgccacctgac

6. pCL223- pLV-U6-[hSNCA gRNA 2]-EFSNC-dSpCas9-DNMT3a-P2A-PURO-WPRE (THERAPUTIC VECTOR)

ctagttattaatagtaatcaattacggggtcattagttcatagcccatatatggagttccgcgttacataacttacggtaaatggcccgcctggct
gaccgcccaacgacccccgcccattgacgtcaataatgacgtatgttcccatagtaacgccaatagggactttccattgacgtcaatgggtg
gagtatttacggtaaactgcccacttggcagtacatcaagtgtatcatatgccaagtacgccccctattgacgtcaatgacggtaaatggccc
gcctggcattatgcccagtacatgaccttatgggactttcctacttggcagtacatctacgtattagtcatcgctattaccatggtgatgcggtttt
ggcagtacatcaatgggcgtggatagcggtttgactcacggggatttccaagtctccaccccattgacgtcaatgggagtttgttttggcacc
aaaatcaacgggactttccaaaatgtcgtaacaactccgccccattgacgcaaatgggcggtaggcgtgtacggtgggaggtctatataag
cagcgcgttttgcctgtactgggtctctctggttagaccagatctgagcctgggagctctctggctaactagggaacccactgcttaagcctca
ataaagcttgccttgagtgcttcaagtagtgtgtgcccgtctgttgtgtgactctggtaactagagatccctcagacccttttagtcagtgtggaa
aatctctagcagtggcgcccgaacagggacttgaaagcgaaagggaaaccagaggagctctctcgacgcaggactcggcttgctgaagc
gcgcacggcaagaggcgaggggcggcgactggtgagtacgccaaaaattttgactagcggaggctagaaggagagagatgggtgcga
gagcgtcagtattaagcgggggagaattagatcgcgatgggaaaaaattcggttaaggccagggggaaagaaaaaatataaattaaaaca
tatagtatgggcaagcagggagctagaacgattcgcagttaatcctggcctgttagaaacatcagaaggctgtagacaaatactgggacag
ctacaaccatcccttcagacaggatcagaagaacttagatcattatataatacagtagcaaccctctattgtgtgcatcaaaggatagagataa
aagacaccaaggaagctttagacaagatagaggaagagcaaaacaaaagtaagaccaccgcacagcaagcggccgctgatcttcagac
ctggaggaggagatatgagggacaattggagaagtgaattatataaatataaagtagtaaaaattgaaccattaggagtagcacccaccaa
ggcaaagagaagagtggtgcagagagaaaaaagagcagtgggaataggagctttgttccttgggttcttgggagcagcaggaagcactat
gggcgcagcgtcaatgacgctgacggtacaggccagacaattattgtctggtatagtgcagcagcagaacaatttgctgagggctattgag
gcgcaacagcatctgttgcaactcacagtctggggcatcaagcagctccaggcaagaatcctggctgtggaaagatacctaaaggatcaa
cagctcctggggatttggggttgctctggaaaactcatttgcaccactgctgtgccttggaatgctagttggagtaataaatctctggaacagat
ttggaatcacacgacctggatggagtgggacagagaaattaacaattacacaagcttaatacactccttaattgaagaatcgcaaaaccagc
aagaaaagaatgaacaagaattattggaattagataaatgggcaagtttgtggaattggtttaacataacaaattggctgtggtatataaaattat
tcataatgatagtaggaggcttggtaggtttaagaatagtttttgctgtactttctatagtgaatagagttaggcagggatattcaccattatcgttt
cagacccacctcccaaccccgaggggacccgacaggcccgaaggaatagaagaagaaggtggagagagagacagagacagatccat
tcgattagtgaacggatcggcactgcgtgcgccaattctgcagacaaatggcagtattcatccacaattttaaaagaaaaggggggattggg
gggtacagtgcaggggaaagaatagtagacataatagcaacagacatacaaactaaagaattacaaaaacaaattacaaaaattcaaaattt
tcgggtttattacagggacagcagagatccagtttggTTAATTAATGGGCGGGACGTTAACGGGGCGGAAC
GGTACCgagggcctatttcccatgattccttcatatttgcatatacgatacaaggctgttagagagataattagaattaatttgactgtaaac
acaaagatattagtacaaaatacgtgacgtagaaagtaataatttcttgggtagtttgcagttttaaaattatgttttaaaatggactatcatatgctt

accgtaacttgaaagtatttcgatttcttggctttatatatcttGTGGAAAGGACGAAAcaccgCTGCTCAGGGTAG
ATAGCTGgTTTtagtactctggaaacagaatctactaaaacaaggcaaaatgccgtgtttatctcgtcaacttgttggcgagaTTT
TTTgaattcgctagctaggtcttgaaaggagtgggaattggctccggtgcccgtcagtgggcagagcgcacatcgcccacagtccccga
gaagttggggggaggggtcggcaattgatccggtgcctagagaaggtggcgcggggtaaactgggaaagtgatgtcgtgtactggctcc
gcctttttcccgagggtgggggagaaccgtatataagtgcagtagtcgccgtgaacgttctttttcgcaacgggtttgccgccagaacacag
gaccggtgccaccatgGACTACAAAGACCATGACGGTGATTATAAAGATCATGACATaGATT
ACAAGGATGACGATGACAAGggtggatcgccaaagaaaaagaggaaagtcgccccaaagaagaagcggaaggtcg
gtatccacggagtcccagcagccaagcggaactacatcctgggcctggccatcggcatcaccagcgtgggctacggcatcatcgactac
gagacacgggacgtgatcgatgccggcgtgcggctgttcaaagaggccaacgtggaaaacaacgagggcaggcggagcaagagagg
cgccagaaggctgaagcggcggaggcggcatagaatccagagagtgaagaagctgctgttcgactacaacctgctgaccgaccacagc
gagctgagcggcatcaacccctacgaggccagagtgaagggcctgagccagaagctgagcgaggaagagttctctgccgccctgctgc
acctggccaagagaagaggcgtgcacaacgtgaacgaggtggaagaggacaccggcaacgagctgtccaccaaagagcagatcagc
cggaacagcaaggccctggaagagaaatacgtggccgaactgcagctggaacggctgaagaaagacggcgaagtgcggggcagcat
caacagattcaagaccagcgactacgtgaaagaagccaaacagctgctgaaggtgcagaaggcctaccaccagctggaccagagcttca
tcgacacctacatcgacctgctggaaacccggcggacctactatgagggacctggcgagggcagccccttcggctggaaggacatcaaa
gaatggtacgagatgctgatgggccactgcacctacttccccgaggaactgcggagcgtgaagtacgcctacaacgccgacctgtacaac
gccctgaacgacctgaacaatctcgtgatcaccagggacgagaacgagaagctggaatattacgagaagttccagatcatcgagaacgtg
ttcaagcagaagaagaagcccaccctgaagcagatcgccaaagaaatcctcgtgaacgaagaggatattaagggctacagagtgaccag
caccggcaagcccgagttcaccaacctgaaggtgtaccacgacatcaaggacattaccgcccggaaagagattattgagaacgccgagc
tgctggatcagattgccaagatcctgaccatctaccagagcagcgaggacatccaggaagaactgaccaatctgaactccgagctgaccc
aggaagagatcgagcagatctctaatctgaagggctataccggcacccacaacctgagcctgaaggccatcaacctgatcctggacgagc
tgtggcacaccaacgacaaccagatcgctatcttcaaccggctgaagctggtgcccaagaaggtggacctgtcccagcagaaagagatcc
ccaccaccctggtggacgacttcatcctgagccccgtcgtgaagagaagcttcatccagagcatcaaagtgatcaacgccatcatcaagaa
gtacggcctgcccaacgacatcattatcgagctggcccgcgagaagaactccaaggacgcccagaaaatgatcaacgagatgcagaagc
ggaaccggcagaccaacgagcggatcgaggaaatcatccggaccaccggcaaagagaacgccaagtacctgatcgagaagatcaagc
tgcacgacatgcaggaaggcaagtgcctgtacagcctggaagccatccctctggaagatctgctgaacaaccccttcaactatgaggtgga
ccacatcatccccagaagcgtgtccttcgacaacagcttcaacaacaaggtgctcgtgaagcaggaagaagccagcaagaagggcaacc
ggaccccattccagtacctgagcagcagcgacagcaagatcagctacgaaaccttcaagaagcacatcctgaatctggccaagggcaag
ggcagaatcagcaagaccaagaaagagtatctgctggaagaacgggacatcaacaggttctccgtgcagaaagacttcatcaaccggaa
cctggtggataccagatacgccaccagaggcctgatgaacctgctgcggagctacttcagagtgaacaacctggacgtgaaagtgaagtc
catcaatggcggcttcaccagctttctgcggcggaagtggaagtttaagaaagagcggaacaaggggtacaagcaccacgccgaggacg
ccctgatcattgccaacgccgatttcatcttcaaagagtggaagaaactggacaaggccaaaaaagtgatggaaaaccagatgttcgagga
aaagcaggccgagagcatgcccgagatcgaaaccgagcaggagtacaaagagatcttcatcaccccccaccagatcaagcacattaag
gacttcaaggactacaagtacagccaccgggtggacaagaagcctaatagagagctgattaacgacaccctgtactccacccggaagga
cgacaagggcaacaccctgatcgtgaacaatctgaacggcctgtacgacaaggacaatgacaagctgaaaaagctgatcaacaagagcc
ccgaaaagctgctgatgtaccaccacgacccccagacctaccagaaactgaagctgattatggaacagtacggcgacgagaagaatccc
ctgtacaagtactacgaggaaaccgggaactacctgaccaagtactccaaaaaggacaacggccccgtgatcaagaagattaagtattac
ggcaacaaactgaacgcccatctggacatcaccgacgactaccccaacagcagaaacaaggtcgtgaagctgtccctgaagccctacag
attcgacgtgtacctggacaatggcgtgtacaagttcgtgaccgtgaagaatctggatgtgatcaaaaaagaaaactactacgaagtgaata
gcaagtgctatgaggaagctaagaagctgaagaagatcagcaaccaggccgagtttatcgcctccttctacaacaacgatctgatcaagat
caacggcgagctgtatagagtgatcggcgtgaacaacgacctgctgaaccggatcgaagtgaacatgatcgacatcacctaccgcgagta
cctggaaaacatgaacgacaagaggccccccaggatcattaagacaatcgcctccaagacccagagcattaagaagtacagcacagaca
ttctgggcaacctgtatgaagtgaaatctaagaagcaccctcagatcatcaaaaagggcaaaaggccggcggccacgaaaaaggcGcT
CGAGGGCGGAGGCGGGAGCGGATCCCCCTCCCGGCTCCAGATGttcttcgctaataaccacgacc
aggaatttgaccctccaaaggtttacccacctgtcccagctgagaagaggaagcccatccgggtgctgtctctctttgatggaatcgctacag
ggctcctggtgctgaaggacttgggcattcaggtggaccgctacattgcctcggaggtgtgtgaggactccatcacggtgggcatggtgcg

gcaccaggggaagatcatgtacgtcggggacgtccgcagcgtcacacagaagcatatccaggagtggggcccattcgatctggtgattg
ggggcagtccctgcaatgacctctccatcgtcaaccctgctcgcaagggcctctacgagggcactggccggctcttctttgagttctaccgc
ctcctgcatgatgcgcggcccaaggagggagatgatcgcccccttcttctggctctttgagaatgtggtggccatgggcgttagtgacaagag
ggacatctcgcgatttctcgagtccaaccctgtgatgattgatgccaaagaagtgtcagctgcacacagggcccgctacttctggggtaacct
tcccggtatgaacaggccgttggcatccactgtgaatgataagctggagctgcaggagtgtctggagcatggcaggatagccaagttcagc
aaagtgaggaccattactacgaggtcaaactccataaagcagggcaaaGACCAGCATTTTCCTGTGTTCATGAAT
GAGAAAGAGgacatcttatggtgcactgaaatggaaagggtatttggtttcccagtccactatactgacgtgtccaacatgagccgct
tggcgaggcagagactgctgggccggtcatggagcgtgccagtcatccgccacctcttcgctcCGCTGAAGGAGTATTTT
GCGTGTGTGtccggccggcccggatccGGCGCAACAAACTTCTCTCTGCTGAAACAAGCCGGA
GATGTCGAAGAGAATCCTGGACCGACCGAGTACAAGCCCACGGTGCGCCTCGCCAC
CCGCGACGACGTCCCCAGGGCCGTACGCACCCTCGCCGCCGCGTTCGCCGACTACCC
CGCCACGCGCCACACCGTCGATCCGGACCGCCACATCGAGCGGGTCACCGAGCTGC
AAGAACTCTTCCTCACGCGCGTCGGGCTCGACATCGGCAAGGTGTGGGTCGCGGAC
GACGGCGCCGCGGTGGCGGTCTGGACCACGCCGGAGAGCGTCGAAGCGGGGGCGG
TGTTCGCCGAGATCGGCCCGCGCATGGCCGAGTTGAGCGGTTCCCGGCTGGCCGCGC
AGCAACAGATGGAAGGCCTCCTGGCGCCGCACCGGCCCAAGGAGCCCGCGTGGTTC
CTGGCCACCGTCGGAGTCTCGCCCGACCACCAGGGCAAGGGTCTGGGCAGCGCCGT
CGTGCTCCCCGGAGTGGAGGCGGCCGAGCGCGCCGGGGTGCCCGCCTTCCTGGAGA
CCTCCGCGCCCCGCAACCTCCCCTTCTACGAGCGGCTCGGCTTCACCGTCACCGCCG
ACGTCGAGGTGCCCGAAGGACCGCGCACCTGGTGCATGACCCGCAAGCCCGGTGCC
TGAACGCGTTAAGTCGACAATCAACCTCTGGATTACAAAATTTGTGAAAGATTGACT
GGTATTCTTAACTATGTTGCTCCTTTTACGCTATGTGGATACGCTGCTTTAATGCCTT
TGTATCATGCTATTGCTTCCCGTATGGCTTTCATTTTCTCCTCCTTGTATAAATCCTGG
TTGCTGTCTCTTTATGAGGAGTTGTGGCCCGTTGTCAGGCAACGTGGCGTGGTGTGC
ACTGTGTTTGCTGACGCAACCCCCACTGGTTGGGGCATTGCCACCACCTGTCAGCTC
CTTTCCGGGACTTTCGCTTTCCCCCTCCCTATTGCCACGGCGGAACTCATCGCCGCCT
GCCTTGCCCGCTGCTGGACAGGGGCTCGGCTGTTGGGCACTGACAATTCCGTGGTGT
TGTCGGGGAAATCATCGTCCTTTCCTTGGCTGCTCGCCTGTGTTGCCACCTGGATTCT
GCGCGGGACGTCCTTCTGCTACGTCCCTTCGGCCCTCAATCCAGCGGACCTTCCTTCC
CGCGGCCTGCTGCCGGCTCTGCGGCCTCTTCCGCGTCTTCGCCTTCGCCCTCAGACG
AGTCGGATCTCCCTTTGGGCCGCCTCCCCGCGTCGACTTTAAGACCAATGACTTACA
AGGCAGCTGTAGATCTTAGCCACTTTTTAAAAGAAAAGGGGGGACTGGAAGGGCTA
ATTCACTCCCAACGAAGACAAGATCTGCTTTTTGCTTGTACTGGGTCTCTCTGGTTAG
ACCAGATCTGAGCCTGGGAGCTCTCTGGCTAACTAGGGAACCCACTGCTTAAGCCTC
AATAAAGCTTGCCTTGAGTGCTTCAAGTAGTGTGTGCCCGTCTGTTGTGTGACTCTG
GTAACTAGAGATCCCTCAGACCCTTTTAGTCAGTGTGGAAAATCTCTAGCAGggcccgttt
aaacccgctgatcagcctcgactgtgccttctagttgccagccatctgttgtttgcccctcccccgtgccttccttgaccctggaaggtgccact
cccactgtcctttcctaataaaatgaggaaattgcatcgcattgtctgagtaggtgtcattctattctggggggtggggtggggcaggacagc
aagggggaggattgggaagacaatagcaggcatgctggggatgcggtgggctctatggcttctgaggcggaaagaaccagctggggct
ctagggggtatccccacgcgccctgtagcggcgcattaagcgcggcgggtgtggtggttacgcgcagcgtgaccgctacacttgccagc
gccctagcgcccgctcctttcgctttcttcccttcctttctcgccacgttcgccggctttccccgtcaagctctaaatcgggggctccctttaggg
ttccgatttagtgctttacggcacctcgaccccaaaaaacttgattagggtgatggttcacgtagtgggccatcgccctgatagacggtttttcg
ccctttgacgttggagtccacgttctttaatagtggactcttgttccaaactggaacaacactcaaccctatctcggtctattcttttgatttataatg
gttacaaataaagcaatagcatcacaaatttcacaaataaagcatttttttcactgcattctagttgtggtttgtccaaactcatcaatgtatcttatc
atgtctgtataccgtcgacctctagctagagcttggcgtaatcatggtcatagctgtttcctgtgtgaaattgttatccgctcacaattccacacaa
catacgagccggaagcataaagtgtaaagcctggggtgcctaatgagtgagctaactcacattaattgcgttgcgctcactgcccgctttcca

gtcgggaaacctgtcgtgccagctgcattaatgaatcggccaacgcgcggggagaggcggtttgcgtattgggcgctcttccgcttcctcg
ctcactgactcgctgcgctcggtcgttcggctgcggcgagcggtatcagctcactcaaaggcggtaatacggttatccacagaatcagggg
ataacgcaggaaagaacatgtgagcaaaaggccagcaaaaggccaggaaccgtaaaaaggccgcgttgctggcgtttttccataggctc
cgcccccctgacgagcatcacaaaaatcgacgctcaagtcagaggtggcgaaacccgacaggactataaagataccaggcgtttccccct
ggaagctccctcgtgcgctctcctgttccgaccctgccgcttaccggatacctgtccgcctttctcccttcgggaagcgtggcgctttctcata
gctcacgctgtaggtatctcagttcggtgtaggtcgttcgctccaagctgggctgtgtgcacgaaccccccgttcagcccgaccgctgcgcc
ttatccggtaactatcgtcttgagtccaacccggtaagacacgacttatcgccactggcagcagccactggtaacaggattagcagagcgag
gtatgtaggcggtgctacagagttcttgaagtggtggcctaactacggctacactagaagaacagtatttggtatctgcgctctgctgaagcc
agttaccttcggaaaaagagttggtagctcttgatccggcaaacaaaccaccgctggtagcggtggtttttttgtttgcaagcagcagattacg
cgcagaaaaaaaggatctcaagaagatcctttgatcttttctacggggtctgacgctcagtggaacgaaaactcacgttaagggattttggtc
atgagattatcaaaaaggatcttcacctagatccttttaaattaaaaatgaagttttaaatcaatctaaagtatatatgagtaaacttggtctgacag
ttaccaatgcttaatcagtgaggcacctatctcagcgatctgtctatttcgttcatccatagttgcctgactccccgtcgtgtagataactacgata
cgggagggcttaccatctggccccagtgctgcaatgataccgcgagacccacgctcaccggctccagatttatcagcaataaaccagcca
gccggaagggccgagcgcagaagtggtcctgcaactttatccgcctccatccagtctattaattgttgccgggaagctagagtaagtagttc
gccagttaatagtttgcgcaacgttgttgccattgctacaggcatcgtggtgtcacgctcgtcgtttggtatggcttcattcagctccggttccca
acgatcaaggcgagttacatgatcccccatgttgtgcaaaaaagcggttagctccttcggtcctccgatcgttgtcagaagtaagttggccgc
agtgttatcactcatggttatggcagcactgcataattctcttactgtcatgccatccgtaagatgcttttctgtgactggtgagtactcaaccaag
tcattctgagaatagtgtatgcggcgaccgagttgctcttgcccggcgtcaatacgggataataccgcgccacatagcagaactttaaaagtg
ctcatcattggaaaacgttcttcggggcgaaaactctcaaggatcttaccgctgttgagatccagttcgatgtaacccactcgtgcacccaact
gatcttcagcatcttttactttcaccagcgtttctgggtgagcaaaaacaggaaggcaaaatgccgcaaaaaagggaataagggcgacacg
gaaatgttgaatactcatactcttcctttttcaatattattgaagcatttatcagggttattgtctcatgagcggatacatatttgaatgtatttagaaa
aataaacaaataggggttccgcgcacatttccccgaaaagtgccacctgacgtcgacggatcgggagatctcccgatcccctatggtgcac
tctcagtacaatctgctctgatgccgcatagttaagccagtatctgctccctgcttgtgtgttggaggtcgctgagtagtgcgcgagcaaaattt
aagctacaacaaggcaaggcttgaccgacaattgcatgaagaatctgcttagggttaggcgttttgcgctgcttcgcgatgtacgggccaga
tatacgcgctgacattgattatt

7. **BK1837- pLV-CMV-SNCAintron1-AlphaSyn-d2EGFP-P2A-NLuc-SV40-Puro-WPRE (pCL35)**

gcacttttcggggaaatgtgcgcggaacccctatttgtttatttttctaaatacattcaaatatgtatccgctcatgagacaataaccctgataaat
gcttcaataatattgaaaaaggaagagtatgagtattcaacatttccgtgtcgcccttattcccttttttgcggcattttgccttcctgtttttgctcac
ccagaaacgctggtgaaagtaaaagatgctgaagatcagttgggtgcacgagtgggttacatcgaactggatctcaacagcggtaagatcc
ttgagagttttcgccccgaagaacgttttccaatgatgagcacttttaaagttctgctatgtggcgcggtattatcccgtattgacgccgggcaa
gagcaactcggtcgccgcatacactattctcagaatgacttggttgagtactcaccagtcacagaaaagcatcttacggatggcatgacagta
agagaattatgcagtgctgccataaccatgagtgataacactgcggccaacttacttctgacaacgatcggaggaccgaaggagctaaccg
cttttttgcacaacatgggggatcatgtaactcgccttgatcgttgggaaccggagctgaatgaagccataccaaacgacgagcgtgacacc
acgatgcctgtagcaatggcaacaacgttgcgcaaactattaactggcgaactacttactctagcttcccggcaacaattaatagactggatg
gaggcggataaagttgcaggaccacttctgcgctcggcccttccggctggctggtttattgctgataaatctggagccggtgagcgtgggtc
tcgcggtatcattgcagcactggggccagatggtaagccctcccgtatcgtagttatctacacgacggggagtcaggcaactatggatgaa
cgaaatagacagatcgctgagataggtgcctcactgattaagcattggtaactgtcagaccaagtttactcatatatactttagattgatttaaaa
cttcatttttaatttaaaaggatctaggtgaagatcctttttgataatctcatgaccaaaatcccttaacgtgagttttcgttccactgagcgtcaga
ccccgtagaaaagatcaaaggatcttcttgagatcctttttttctgcgcgtaatctgctgcttgcaaacaaaaaaaccaccgctaccagcggtg
gtttgtttgccggatcaagagctaccaactctttttccgaaggtaactggcttcagcagagcgcagataccaaatactgttcttctagtgtagcc
gtagttaggccaccacttcaagaactctgtagcaccgcctacatacctcgctctgctaatcctgttaccagtggctgctgccagtggcgataa

gtcgtgtcttaccgggttggactcaagacgatagttaccggataaggcgcagcggtcgggctgaacggggggttcgtgcacacagcccag
cttggagcgaacgacctacaccgaactgagatacctacagcgtgagctatgagaaagcgccacgcttcccgaagggagaaaggcggac
aggtatccggtaagcggcagggtcggaacaggagagcgcacgagggagcttccagggggaaacgcctggtatctttatagtcctgtcgg
gtttcgccacctctgacttgagcgtcgatttttgtgatgctcgtcaggggggcggagcctatggaaaaacgccagcaacgcggcctttttacg
gttcctggccttttgctggccttttgctcacatgttctttcctgcgttatcccctgattctgtggataaccgtattaccgcctttgagtgagctgatac
cgctcgccgcagccgaacgaccgagcgcagcgagtcagtgagcgaggaagcggaagagcgcccaatacgcaaaccgcctctccccg
cgcgttggccgattcattaatgcagctggcacgacaggtttcccgactggaaagcgggcagtgagcgcaacgcaattaatgtgagttagct
cactcattaggcaccccaggctttacactttatgcttccggctcgtatgttgtgtggaattgtgagcggataacaatttcacacaggaaacagct
atgaccatgattacgccaagcgcgcaattaaccctcactaaagggaacaaaagctggagctgcaagcttaatgtagtcttatgcaatactctt
gtagtcttgcaacatggtaacgatgagttagcaacatgccttacaaggagagaaaaagcaccgtgcatgccgattggtggaagtaaggtgg
tacgatcgtgccttattaggaaggcaacagacgggtctgacatggattggacgaaccactgaattgccgcattgcagagatattgtatttaagt
gcctagctcgatacataaacgggtctctctggttagaccagatctgagcctgggagctctctggctaactagggaacccactgcttaagcctc
aataaagcttgccttgagtgcttcaagtagtgtgtgcccgtctgttgtgtgactctggtaactagagatccctcagacccttttagtcagtgtgga
aaatctctagcagtggcgcccgaacagggacttgaaagcgaaagggaaaccagaggagctctctcgacgcaggactcggcttgctgaag
cgcgcacggcaagaggcgaggggcggcgactggtgagtacgccaaaaattttgactagcggaggctagaaggagagagatgggtgcg
agagcgtcagtattaagcgggggagaattagatcgcgatgggaaaaaattcggttaaggccagggggaaagaaaaaatataaattaaaac
atatagtatgggcaagcagggagctagaacgattcgcagttaatcctggcctgttagaaacatcagaaggctgtagacaaatactgggaca
gctacaaccatcccttcagacaggatcagaagaacttagatcattatataatacagtagcaaccctctattgtgtgcatcaaaggatagagata
aaagacaccaaggaagctttagacaagatagaggaagagcaaaacaaaagtaagaccaccgcacagcaagcggccgctgatcttcaga
cctggaggaggagatatgagggacaattggagaagtgaattatataaatataaagtagtaaaaattgaaccattaggagtagcacccaccaa
ggcaaagagaagagtggtgcagagagaaaaaagagcagtgggaataggagctttgttccttgggttcttgggagcagcaggaagcactat
gggcgcagcgtcaatgacgctgacggtacaggccagacaattattgtctggtatagtgcagcagcagaacaatttgctgagggctattgag
gcgcaacagcatctgttgcaactcacagtctggggcatcaagcagctccaggcaagaatcctggctgtggaaagatacctaaaggatcaa
cagctcctggggatttggggttgctctggaaaactcatttgcaccactgctgtgccttggaatgctagttggagtaataaatctctggaacagat
ttggaatcacacgacctggatggagtgggacagagaaattaacaattacacaagcttaatacactccttaattgaagaatcgcaaaaccagc
aagaaaagaatgaacaagaattattggaattagataaatgggcaagtttgtggaattggtttaacataacaaattggctgtggtatataaaattat
tcataatgatagtaggaggcttggtaggtttaagaatagtttttgctgtactttctatagtgaatagagttaggcagggatattcaccattatcgttt
cagacccacctcccaaccccgaggggacccgacaggcccgaaggaatagaagaagaaggtggagagagagacagagacagatccat
tcgattagtgaacggatctcgacggtatcggttaacttttaaaagaaaaggggggattggggggtacagtgcaggggaaagaatagtagac
ataatagcaacagacatacaaactaaagaattacaaaaacaaattacaaaattcaaaattttatCGATTTAATTAACGCGTTA
CATAACTTACGGTAAATGGCCCGCCTGGCTGACCGCCCAACGACCCCCGCCCATTGAC
GTCAATAATGACGTATGTTCCCATAGTAACGCCAATAGGGACTTTCCATTGACGTCAAT
GGGTGGAGTATTTACGGTAAACTGCCCACTTGGCAGTACATCAAGTGTATCATATGCC
AAGTACGCCCCCTATTGACGTCAATGACGGTAAATGGCCCGCCTGGCATTATGCCCAG
TACATGACCTTATGGGACTTTCCTACTTGGCAGTACATCTACGTATTAGTCATCGCTATT
ACCATGGTCGAGGTGAGCCCCACGTTCTGCTTCACTCTCCCCATCTCCCCCCCCTCCC
CACCCCCAATTTTGTATTTATTTATTTTTTAATTATTTTGTGCAGCGATGGGGGCGGGGG
GGGGGGGGGGCGCGCGCCAGGCGGGGCGGGGCGGGGCGAGGGGCGGGGCGGGG
CGAGGCGGAGAGGTGCGGCGGCAGCCAATCAGAGCGGCGCGCTCCGAAAGTTTCCT
TTTATGGCGAGGCGGCGGCGGCGGCGGCCCTATAAAAAGCGAAGCGCGCGGCGGGC

Gcgggcgtctttggaaatcctggagaacgccggatgggagacgaatggtcgtgggcaccgggagggggtggtgctgccatgaggacc
cgctgggccaggtctctgggaggtgagtacttgtccctttggggagcctaaggaaagagacttgacctggctttcgtcctgcttctgatattcc
cttctccacaagggctgagagattaggctgcttctccgggatccgcttttccccgggaaacgcgaggatgctccatggagcgtgagcatcc
aacttttctctcacataaaatctgtctgcccgctctcttggtttttctctgtaaagtaagcaagctgcgtttggcaaataatgaaatggaagtgcaa
ggaggccaagtcaacaggtggtaacgggttaacaagtgctggcgcggggtccgctagggtggaggctgagaacgccccctcgggtggc
tggcgcggggttggagacggcccgcgagtgtgagcggcgcctgctcagggtagatagctgagggcggATGgatgtattcatgaaagg
actttcaaaggccaaggagggagttgtggctgctgctgagaaaaccaaacagggtgtggcagaagcagcaggaaagacaaaagagggt
gttctctatgtaggctccaaaaccaaggagggagtggtgcatggtgtggcaacagtggctgagaagaccaaagagcaagtgacaaatgtt
ggaggagcagtggtgacgggtgtgacagcagtagcccagaagacagtggagggagcagggagcattgcagcagccactggctttgtca
aaaaggaccagttgggcaagaatgaagaaggagccccacaggaaggaattctggaagatatgcctgtggatcctgacaatgaggcttatg
aaatgccttctgaggaagggtatcaagactacgaacctgaagAGGAGGAGAGTGACTTAATTAAAccggtcgccacc
atggtgagcaagggcgaggagctgttcaccggggtggtgcccatcctggtcgagctggacggcgacgtaaacggccacaagttcagcgt
gtccggcgagggcgagggcgatgccacctacggcaagctgaccctgaagttcatctgcaccaccggcaagctgcccgtgccctggccc
accctcgtgaccaccctgacctacggcgtgcagtgcttcagccgctaccccgaccacatgaagcagcacgacttcttcaagtccgccatgc
ccgaaggctacgtccaggagcgcaccatcttcttcaaggacgacggcaactacaagacccgcgccgaggtgaagttcgagggcgacac
cctggtgaaccgcatcgagctgaagggcatcgacttcaaggaggacggcaacatcctggggcacaagctggagtacaactacaacagcc
acaacgtctatatcatggccgacaagcagaagaacggcatcaaggtgaacttcaagatccgccacaacatcgaggacggcagcgtgcag
ctcgccgaccactaccagcagaacacccccatcggcgacggccccgtgctgctgcccgacaaccactacctgagcacccagtccgccct
gagcaaagaccccaacgagaagcgcgatcacatggtcctgctggagttcgtgaccgccgccgggatcactctcggcatggacgagctgt
acaagaagcttagccatggcttcccgccggaggtggaggagcaggatgatggcacgctgcccatgtcttgtgcccaggagagcgggatg
gaccgtcaccctgcagcctgtgcttctgctaggatcaatgtgGaggAggGcgGCTCgAGAGCCACGAACTTCTCT
CTGTTAAAGCAAGCAGGAGACGTGGAAGAAAACCCCGGTCCCGCCACCATGGTCTT
CACACTCGAAGATTTCGTTGGGGACTGGCGACAGACAGCCGGCTACAACCTGGACC
AAGTCCTTGAACAGGGAGGTGTGTCCAGTTTGTTTCAGAATCTCGGGGTGTCCGTAA
CTCCGATCCAAAGGATTGTCCTGAGCGGTGAAAATGGGCTGAAGATCGACATCCATG
TCATCATCCCGTATGAAGGTCTGAGCGGCGACCAAATGGGCCAGATCGAAAAAATTTT
TAAGGTGGTGTACCCTGTGGATGATCATCACTTTAAGGTGATCCTGCACTATGGCACA
CTGGTAATCGACGGGGTTACGCCGAACATGATCGACTATTTCGGACGGCCGTATGAAG
GCATCGCCGTGTTCGACGGCAAAAAGATCACTGTAACAGGGACCCTGTGGAACGGC
AACAAAATTATCGACGAGCGCCTGATCAACCCCGACGGCTCCCTGCTGTTCCGAGTA
ACCATCAACGGAGTGACCGGCTGGCGGCTGTGCGAACGCATTCTGGCGTAAGtcgaCcat
gcatctagacccagcggCgCGcCCCTGTGGAATGTGTGTCAGTTAGGGTGTGGAAAGTCCCCAG
GCTCCCCAGCAGGCAGAAGTATGCAAAGCATGCATCTCAATTAGTCAGCAACCAGGT
GTGGAAAGTCCCCAGGCTCCCCAGCAGGCAGAAGTATGCAAAGCATGCATCTCAATT
AGTCAGCAACCATAGTCCCGCCCCTAACTCCGCCCATCCCGCCCCTAACTCCGCCCAG
TTCCGCCCATTCTCCGCCCCATGGCTGACTAATTTTTTTTATTTATGCAGAGGCCGAGG
CCGCCTCGGCCTCTGAGCTATTCCAGAAGTAGTGAGGAGGCTTTTTTGGAGGCCTAG
GCTTTTGCAAAAAGCTTACCATGACCGAGTACAAGCCCACGGTGCGCCTCGCCACCC
GCGACGACGTCCCCAGGGCCGTACGCACCCTCGCCGCCGCGTTCGCCGACTACCCCG
CCACGCGCCACACCGTCGATCCGGACCGCCACATCGAGCGGGTCACCGAGCTGCAA

GAACTCTTCCTCACGCGCGTCGGGCTCGACATCGGCAAGGTGTGGGTCGCGGACGAC
GGCGCCGCGGTGGCGGTCTGGACCACGCCGGAGAGCGTCGAAGCGGGGGCGGTGTT
CGCCGAGATCGGCCCGCGCATGGCCGAGTTGAGCGGTTCCCGGCTGGCCGCGCAGC
AACAGATGGAAGGCCTCCTGGCGCCGCACCGGCCCAAGGAGCCCGCGTGGTTCCTG
GCCACCGTCGGCGTCTCGCCCGACCACCAGGGCAAGGGTCTGGGCAGCGCCGTCGT
GCTCCCCGGAGTGGAGGCGGCCGAGCGCGCCGGGGTGCCCGCCTTCCTGGAGACCT
CCGCGCCCCGCAACCTCCCCTTCTACGAGCGGCTCGGCTTCACCGTCACCGCCGACG
TCGAGGTGCCCGAAGGACCGCGCACCTGGTGCATGACCCGCAAGCCCGGTGCCTGAc
gGgcgcgccgAATTCGACAATCAACCTCTGGATTACAAAATTTGTGAAAGATTGACTGGT
ATTCTTAACTATGTTGCTCCTTTTACGCTATGTGGATACGCTGCTTTAATGCCTTTGTAT
CATGCTATTGCTTCCCGTATGGCTTTCATTTTCTCCTCCTTGTATAAATCCTGGTTGCTG
TCTCTTTATGAGGAGTTGTGGCCCGTTGTCAGGCAACGTGGCGTGGTGTGCACTGTGT
TTGCTGACGCAACCCCCACTGGTTGGGGCATTGCCACCACCTGTCAGCTCCTTTCCG
GGACTTTCGCTTTCCCCCTCCCTATTGCCACGGCGGAACTCATCGCCGCCTGCCTTGC
CCGCTGCTGGACAGGGGCTCGGCTGTTGGGCACTGACAATTCCGTGGTGTTGTCGGG
GAAATCATCGTCCTTTCCTTGGCTGCTCGCCTGTGTTGCCACCTGGATTCTGCGCGGG
ACGTCCTTCTGCTACGTCCCTTCGGCCCTCAATCCAGCGGACCTTCCTTCCCGCGGCC
TGCTGCCGGCTCTGCGGCCTCTTCCGCGTCTTCGCCTTCGCCCTCAGACGAGTCGGAT
CTCCCTTTGGGCCGCCTCCCCGCGTCGACTTTAAGACCAATGACTTACAAGGTACctttaa
gaccaatgacttacaaggcagctgtagatcttagccactttttaaaagaaaaggggggactggaagggctaattcactcccaacgaagaca
agatctgctttttgcttgtactgggtctctctggttagaccagatctgagcctgggagctctctggctaactagggaacccactgcttaagcctc
aataaagcttgccttgagtgcttcaagtagtgtgtgcccgtctgttgtgtgactctggtaactagagatccctcagacccttttagtcagtgtgga
aaatctctagcagtagtagttcatgtcatcttattattcagtatttataacttgcaaagaaatgaatatcagagagtgagaggaacttgtttattgca
gcttataatggttacaaataaagcaatagcatcacaaatttcacaaataaagcatttttttcactgcattctagttgtggtttgtccaaactcatcaat
gtatcttatcatgtctggctctagctatcccgcccctaactccgcccatcccgcccctaactccgcccagttccgcccattctccgccccatgg
ctgactaattttttttatttatgcagaggccgaggccgcctcggcctctgagctattccagaagtagtgaggaggcttttttggaggcctaggga
cgtacccaattcgccctatagtgagtcgtattacgcgcgctcactggccgtcgttttacaacgtcgtgactgggaaaaccctggcgttaccca
acttaatcgccttgcagcacatccccctttcgccagctggcgtaatagcgaagaggcccgcaccgatcgcccttcccaacagttgcgcagc
ctgaatggcgaatgggacgcgccctgtagcggcgcattaagcgcggcgggtgtggtggttacgcgcagcgtgaccgctacacttgccag
cgccctagcgcccgctcctttcgctttcttcccttcctttctcgccacgttcgccggctttccccgtcaagctctaaatcgggggctccctttagg
gttccgatttagtgctttacggcacctcgaccccaaaaaacttgattagggtgatggttcacgtagtgggccatcgccctgatagacggtttttc
gccctttgacgttggagtccacgttctttaatagtggactcttgttccaaactggaacaacactcaaccctatctcggtctattcttttgatttataa
gggattttgccgatttcggcctattggttaaaaaatgagctgatttaacaaaaatttaacgcgaattttaacaaaatattaacgcttacaatttaggt
g
